# Fin whale acoustic populations present in New Zealand waters: Description of song types, occurrence and seasonality using passive acoustic monitoring

**DOI:** 10.1371/journal.pone.0253737

**Published:** 2021-07-14

**Authors:** Alexandra N. Constaratas, Mark A. McDonald, Kimberly T. Goetz, Giacomo Giorli

**Affiliations:** 1 National Institute of Water and Atmospheric Research, Greta Point, Wellington, New Zealand; 2 Whale Acoustics, Bellvue, Colorado, United States of America; 3 Marine Mammal Laboratory, Alaska Fisheries Science Center, National Marine Fisheries Service, NOAA, Seattle, Washington, United States of America; Wildlife Conservation Society Canada, CANADA

## Abstract

Southern fin whales (*Balaenoptera physalus*) are known to migrate from the Antarctic to mid-latitudes during winter for breeding, but the occurrence and distribution of this species is not well known in the waters around New Zealand. The ‘doublet’ calls are one of the main calls emitted specifically by fin whales and repeated in a regular pattern, which make the acoustic detection of these calls relevant to detect the presence of fin whales. Using a signal processing algorithm to detect ‘doublet’ calls emitted by fin whales, we studied the occurrence, characteristics and seasonality of these ‘doublet’ calls in two regions around New Zealand; Cook Strait in 2016/2017 and offshore Gisborne in 2014/2015. The call detection procedure consisted of binarization of the spectrogram and a cross-correlation between the binarized spectrogram and a template of binarized ‘doublet’ calls spectrogram. A binarization threshold for the data spectrograms and a cross correlation threshold were then determined through multiple trials on a training dataset and a Receiver Operating Characteristics (ROC) curve. Fin whale ‘doublet’ calls occurred on the east side of New Zealand’s Cook Strait during austral winter, specifically in June 2017 and offshore Gisborne in June-August 2014. No ‘doublet’ calls were detected on the west side of Cook Strait. The ‘doublet’ calls’ Inter-Note Interval (INI) was similar in both datasets. However, there was a difference in alternation of the mean frequency for both HF components of ‘doublet’ calls in Cook Strait and Gisborne. As the song types were compared with those previously described in the literature, our findings suggest that some fin whales wintering in New Zealand waters may be part of a broader ‘acoustic population’ whose range extends west to southern Australia and south to Antarctica.

## Introduction

Fin whales (*Balaenoptera physalus*, Linnaeus, 1758) are found throughout most of the world’s oceans, though they are uncommon below latitudes of about 20 degrees and tend not to migrate as far as the polar ice edge [1]. Some populations of fin whales are year-round resident in relatively small areas [2] and are sometimes genetically isolated [3]. Other populations seasonally migrate thousands of kilometers to higher latitudes in summer in pursuit of feeding opportunities [1]. Most of the time, these populations return to subtropical waters in winter to breed [4].

In New Zealand, most of the dedicated visual whale surveys have been conducted in inshore waters. Exceptions are the Japanese whale catcher surveys in the months of October through March between 1965 and 1988 [5]. These surveys showed that fin whales were more abundant near the South Island than the North Island and were particularly abundant off the East Coast of the South Island. Fin whales were spotted in each month when surveys were conducted. Although the distribution and migration of fin whales in New Zealand waters are not well documented, their presence in and around this region (Eastern Subtropical South Pacific in 2012 [6], Great Barrier Island in 1997 [7]; Tasmania in 2006 [8]) has already been documented and their song types briefly described. Sightings of fin whales were reported in the Eastern Subtropical South Pacific [6]. The description of song types was superficial in the Great Barrier Island and in Tasmania because of sampling rate limitations and no description of potential variations over time [7] or no quantitative measurement provided [8].

Most vertebrates use sounds to communicate with conspecifics. Stereotyped calls emitted in a regular sequence are referred to as ‘songs’ [9]. In addition to genetic, morphological, and osteological data, fin whale song types can be used as a proxy to differentiate and identify populations [10–12], which is especially useful when genetic data are insufficient or entirely absent [13]. The term ‘acoustic population’ was defined for whale populations by McDonald, 2006 [7]. While male fin whales are thought to predominantly or exclusively produce songs [14], fin whales are also known to produce non-song calls for social contact or for foraging purpose [15, 16]. Songs are most commonly produced when whales are travelling slowly [17] at depths of 10 to 15 m [18]. Fin whales produce three main calls: 20-Hz calls [19, 20], High Frequency (HF) notes [7, 8, 21, 22], and broadband downsweeps/higher-frequency irregular calls (20 Hz to 90 Hz [19, 23]). The 20-Hz calls can be found in a low-frequency and low-intensity variant, called ‘backbeat’ [24] (note type A in this paper). The ‘backbeat’ can be followed by a ‘classic note’ [25] (note type B in this paper), which is a narrower frequency bandwidth call. The ‘backbeat’ followed by a ‘classic note’ are associated in a regular repeated pattern to form a 20-Hz stereotyped ‘doublet’ [19, 23]. The 20-Hz stereotyped ‘doublet’ are defined by Inter-Note Intervals (INIs) that occur between the two notes composing the ‘doublet’ and between ‘doublets’ (usually between 10 and 30 s respectively [7, 8, 21, 22, 26]). The HF notes are found with the 20-Hz calls produced by fin whales in the southern hemisphere. In the northern hemisphere however, the HF notes can be stand-alone notes or occur not simultaneously with the 20-Hz calls [27]. The HF notes of the southern hemisphere consist of two components: the 1^st^ component and the 2^nd^ component, which are synchronous with each note of the 20-Hz stereotyped ‘doublet’ but at higher frequency energy (between 60 Hz and 100 Hz [7, 8, 21, 22]). This study focuses on the 20-Hz stereotyped ‘doublet’ and on their HF notes. In this paper the 20-Hz stereotyped ‘doublet’ are referred to as ‘doublet’ calls. Furthermore, since this study is located in the southern hemisphere where HF notes are a component of ‘doublet’ calls [7, 8, 21, 22], HF notes are referred to as HF components in this paper. The presence of HF components and INIs may be a way to assign calling animals to known populations or to describe new ‘acoustic populations’ [24]. The third main kind of calls emitted by fin whales, irregular calls, are sporadic and usually spread from 20 Hz to 35 Hz. Irregular calls are difficult to discriminate with other baleen species, and sometimes require visual confirmation. These calls are therefore less appropriate for passive acoustic monitoring of this species [23].

Because fin whale songs are low frequency and can travel long distances underwater; passive acoustic monitoring is an ideal method of studying this species [28]. Songs are thought to be related to mating [14] and vary seasonally [29, 30]. However, researchers have discovered that, unlike blue whale songs whose characteristics remain relatively constant, fin whale song characteristics change across years and within seasons [29, 31] making ‘acoustic populations’ for fin whale more difficult to differentiate than for blue whale [32]. Individual fin whales have also been observed to abruptly switch song type, complicating correlations between song types and ‘acoustic populations’ [33].

Fin whale ‘doublet’ calls have been described in New Zealand waters June through September. However, those recordings lacked sufficient bandwidth and signal-to-noise ratios to fully describe the song types. This may be because fin whales were beyond the continental shelf and too far away from the instruments [7]. North of New Zealand, near Tonga, fin whale ‘doublet’ calls have also been recorded [34], but again, bandwidth limitations prevented a full description of the song types. Southeast of New Zealand, the Antarctic song types is well documented [8, 21, 22].

The main objective of our study is to extend the knowledge of fin whales in New Zealand waters. We chose recording sites where data on fin whales were not collected before, particularly offshore Gisborne and on both sides of Cook Strait. This study provides a description of the characteristics of fin whales’ songs (song types). We assess the occurrence and the seasonality of these songs to observe how they spread across the study sites and time. We also compare the song types of our study with previous studies in New Zealand to investigate whether more than one ‘acoustic population’ exists in New Zealand waters.

## Materials and methods

### Data acquisition

**Fig 1:**
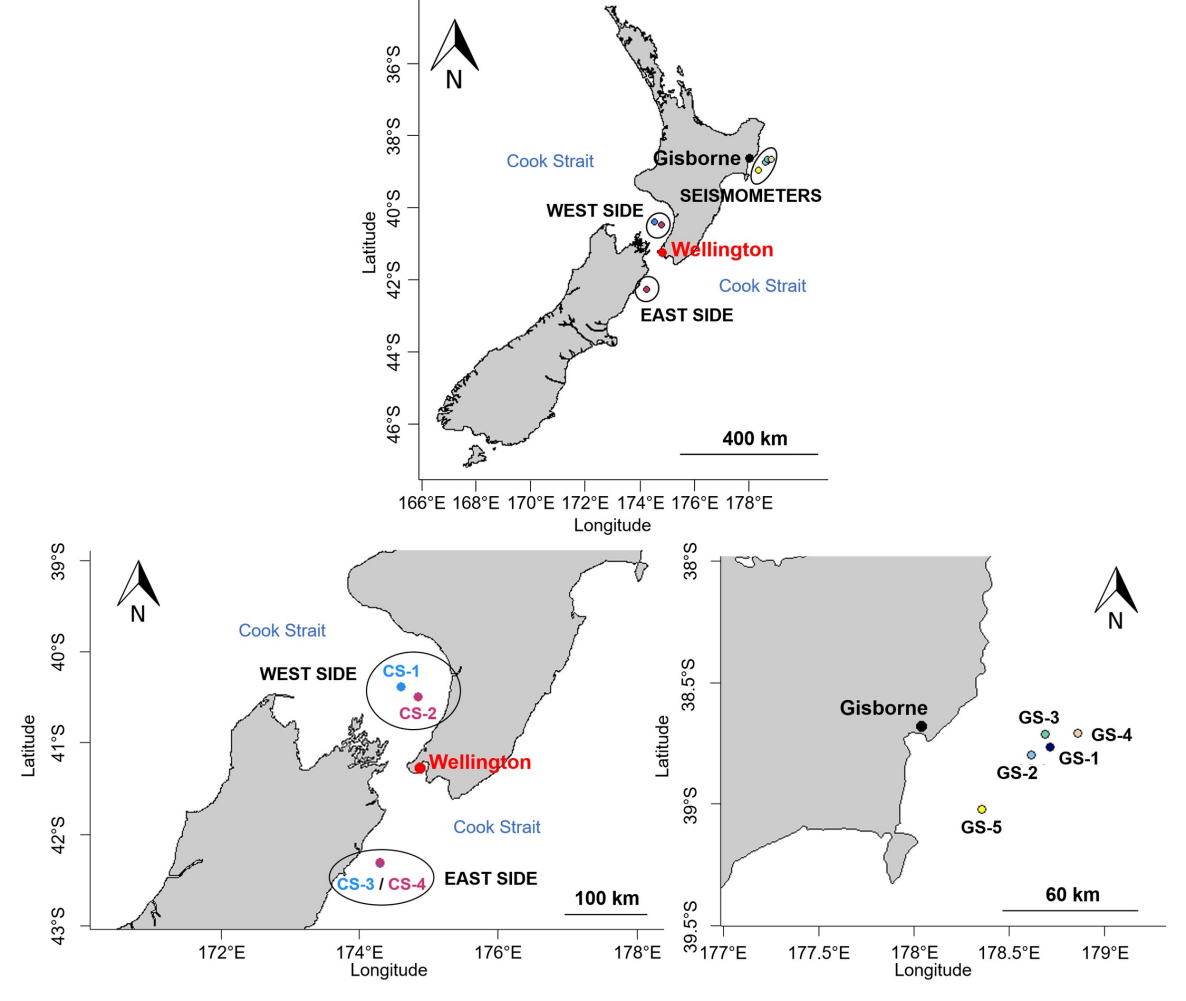
Location and number of the AMARs and the seismometers off New Zealand. Upper: overall map of AMARs and seismometers locations. Lower left: deployment location of the AMARs in the Cook Strait region. Lower right: deployment location of the seismometers offshore Gisborne on the northeast side of the North Island. The deployment were: 2016 (blue) for CS-1 and CS-3; 2017 (pink) for CS-2 and CS-4, and 2014–2015 for GS-1 to GS-5. Maps generated with *maps* package in R (R core Team).

Acoustic data were collected using several instruments deployed in New Zealand waters (Fig 1). Four Autonomous Multichannel Acoustic Recorders (AMAR G3; JASCO Applied Sciences) were deployed on both sides of the Cook Strait and five seismometers (Hikurangi Ocean Bottom Investigation of Tremor and Slow Slip: HOBITSS seismology experiment, Incorporated Research Institutions for Seismology: IRIS data center) were located offshore Gisborne (Fig 1). The AMARs on the west side of Cook Strait (CS) were deployed from early June 2016 to late December 2016 for CS-1 and from mid-February 2017 to early September 2017 for CS-2. On the east side of Cook Strait, AMARs were deployed from late April 2016 to late December 2016 (CS-3) and from mid-February 2017 to early September 2017 (CS-4). The seismometers were deployed offshore Gisborne (GS) from mid-May 2014 to late June 2015. Although there was an AMAR recorder in both west and east side of the Cook Strait recording simultaneously, AMARs (Cook Strait) and seismometers (offshore Gisborne) were not recording at the same time.

All AMARs were equipped with a M36-V35-100 hydrophone (Geospectrum Technologies Inc.) and operated on a duty cycle over 900 seconds: 630 seconds at a sampling rate of 16 kHz, 125 seconds at a sampling rate of 250 kHz, and 145 seconds of sleep. The acoustic recorders were calibrated before the deployment using a pistonphone (GRAS 42AC, GRAS Sound & Vibration A/S, Denmark) and sound levels were converted to dB re 1 μPa^2^/Hz. After calibration, all recorders showed a relatively flat sensitivity of −165 dB re 1 V/μPa between 100 and 10000 Hz. The permits to deploy the AMARs were obtained from the New Zealand Environmental Protection Authority and the Greater Wellington Regional Council.

Due to tidal conditions, CS-2 on the west side of Cook Strait was deployed closer to shore in 2017 (Fig 1). The stations on the east side of the Cook Strait were deployed in deeper waters than the stations on the west side (Table 1).

**Table 1:** Deployments details for the AMARs in the Cook Strait and the seismometers offshore Gisborne.

	Side	Station	Instrument	Latitude	Longitude	Depth (m)	Start date of recording (DD/MM/YYYY)	End date of recording (DD/MM/YYYY)	Number of files analyzed	Total duration recording (days)
Cook Strait	West	CS-1	AMAR	-40.4195	174.5074	110	04/06/2016	21/12/2016	19 040	139
		CS-2	AMAR	-40.5259	174.7571	100	15/02/2017	04/09/2017	17 982	131
	East	CS-3	AMAR	-42.3087	174.2145	1 251	28/04/2016	21/12/2016	19 022	138
		CS-4	AMAR	-42.3071	174.2139	1 200	15/02/2017	08/09/2017	19 092	139
Gisborne	East	GS-1	Seismometer	-38.7459	178.6789	995	13/05/2014	20/06/2015	404	404
		GS-2	Seismometer	-38.7771	178.5835	930	11/05/2014	20/06/2015	406	406
		GS-3	Seismometer	-38.6946	178.6506	1 023	11/05/2014	21/06/2015	405	405
		GS-4	Seismometer	-38.6888	178.8199	1 712	13/05/2014	21/06/2015	405	405
		GS-5	Seismometer	-38.9944	178.3257	1 348	11/02/2014	25/06/2015	378	378

CS: Cook Strait, GS: Gisborne.

About 13 months of ocean bottom seismometer data from the HOBITSS seismology experiment offshore Gisborne New Zealand from 2014–2015 are publicly available at the IRIS data center (five stations: station 1: GS-1; station 2: GS-2; station 3: GS-3; station 4: GS-4; station 5: GS-5). Initial analyses of these data were conducted using the Triton software package and the Raven Pro Interactive Sound analysis software (version 1.5). These data were downloaded using an automated linux shellscript in the miniseed format. Conversion custom software changed the format from the original 24-bit dynamic range to a 16-bit.wav format. In cases where the initial conversion resulted in clipping of signals of particular interest, the conversion was rerun with alternate scaling. The seismometers operated continuously, at a sampling frequency of 200 Hz. There was no data for GS-5 in November 2014 (Table 1). Conversion of seismometer velocities to pressures for comparison to the hydrophone data requires knowledge of the seafloor characteristics, thus we report levels only in relative dB for the Gisborne dataset.

### Detection and peak frequency of ‘doublet’ calls

A fin whale ‘doublet’ calls detector was developed in MATLAB (Mathworks, Natick, MA) using both the Cook Strait and the Gisborne seismometer datasets. Spectrograms from CS-4 on the east side of Cook Strait and GS-1 offshore Gisborne (Fig 1) were visually inspected and the data from these two stations were used as training datasets to develop and test the detection algorithm (S1 Appendix). A 6^th^ order butterworth bandpass filter was applied to retain only bandwidth between 10 Hz and 40 Hz where fin whale ‘doublet’ calls occur [23]. Fin whales ‘doublet’ calls are well documented in the Pacific Ocean and are the predominant calls used by fin whale in lower latitudes [23]. These ‘doublet’ calls were used to train the detector. Detecting a pattern of two notes was chosen to reduce the possibility of falsely detecting other baleen whale species. Additionally as previously mentioned, the other calls emitted by fin whales are irregular, and visual observation is often needed to differentiate fin whales from other baleen whale species [23].

Each fin whale detection was based on the cross-correlation between a ‘doublet’ calls template and the spectrogram of the acoustic data recorded by the AMARs [35]. This approach was successfully used for the stereotyped fin whale calls by other researchers [36]. To create the ‘doublet’ calls template, 10.wav files from the AMARs dataset and 10.wav files from the Gisborne dataset containing fin whale ‘doublet’ calls were selected by visually inspecting spectrograms of the data. These 10.wav files were selected to represent as many various environmental conditions as possible: background noise present and absent, high and low signal-to-noise ratio notes. The spectrogram of each.wav file was obtained by computing the Fast Fourier Transform (FFT) of the data (nfft = sampling frequency, overlap = 50%, Hamming window). The Power Spectral Density (PSD) of each segment of the wav. file, on which the FFT was computed, was returned by the spectrogram function. In the case of AMARs in Cook Strait, levels were calibrated to dB re 1 μPa^2^/Hz (S1 Appendix).

Ten ‘doublet’ calls were manually selected from each of the 10 spectrograms of each dataset. These 10 selected ‘doublet’ calls spectrograms were truncated to only include the bandwidth between 13 Hz and 32 Hz to focus on the ‘doublet’ calls and encompass the two notes of the ‘doublet’ calls. These 10 ‘doublet’ calls were selected as they had high and low signal-to-noise ratio notes, so they could represent many various environmental conditions. This created a dataset of 10 selected ‘doublet’ calls. The first selected ‘doublet’ calls of the ten were then cross-correlated with the other nine. These nine ‘doublet’ calls were selected when the correlation with the first selected ‘doublet’ calls reached the maximum value. In this way, all the ‘doublet’ calls could be precisely synchronized in time and matched to the same time duration. Using this method, the selected ‘doublet’ calls were cut to the same time-frequency size, averaged to compute one single composite ‘doublet’ calls template, and then binarized with a threshold that retained the shape of the ‘doublet’ calls. The binarization approach is commonly used to produce templates of stereotyped calls [34]. In our dataset, we used a binarization threshold of 85 dB (Cook Strait dataset) and -80 relative dB (Gisborne dataset) to produce templates which were manually assessed to keep most of the calls pattern. These threshold values are the dB levels of the spectrogram pixels. If the value of the spectrogram was greater than the thresholds, the pixel value was assigned to 1; if the value was less than the threshold values, the pixel value was assigned to 0. The binarized ‘doublet’ calls template was saved with its frequency bandwidth and was used by the detection algorithm.

**Fig 2:**
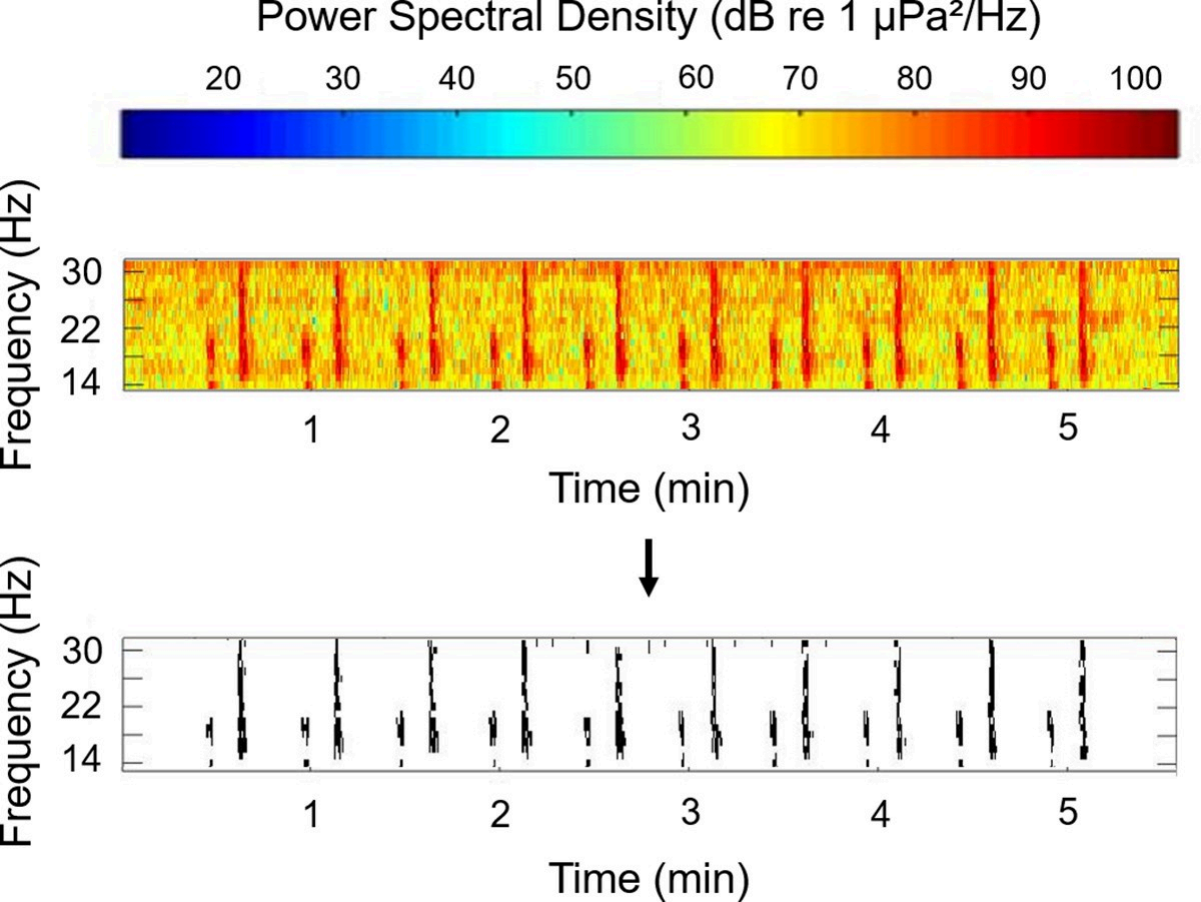
Spectrogram binarization. Colors: spectrogram of one.wav file (here: from the Cook Strait dataset) showing fin whale ‘doublet’ calls. Black and white: binarized spectrogram. Frequency resolution = 1 Hz, time resolution = 0.5 s, binarization thresholds = determined with trials (Cook Strait dataset: 88 dB, Gisborne dataset: -100 relative dB; S1 Appendix).

To detect fin whales in the acoustic data, spectrograms of the.wav files were truncated to include only the frequency bandwidth of the ‘doublet’ calls template. The spectrogram was binarized with a threshold (determined through trials on training datasets) using the same approach applied to the ‘doublet’ calls template binarization (Fig 2).

The fin whale detector required two thresholds: a binarization threshold for the spectrograms of the recorded.wav files and a cross-correlation threshold [26] to assess the match between the ‘doublet’ calls template and the spectrograms (S1 Appendix). ‘Doublet’ calls were detected when the cross-correlation coefficient peaks exceeded the correlation threshold. Fin whale ‘doublet’ calls were considered present in the.wave file when at least one ‘doublet’ call was detected in the.wav file. The detector outputs included the number of ‘doublet’ calls, their average dB levels, the peak frequency of each note and the spectrograms of the detected ‘doublet’ calls. After being validated with the Cook Strait dataset (CS-4 of the East side, Fig 1) and the Gisborne dataset (GS-1, Fig 1), the detector was run on the data collected by the four AMAR stations in Cook Strait (Fig 1) and the five seismometer stations near Gisborne (Fig 1).

**Fig 3:**
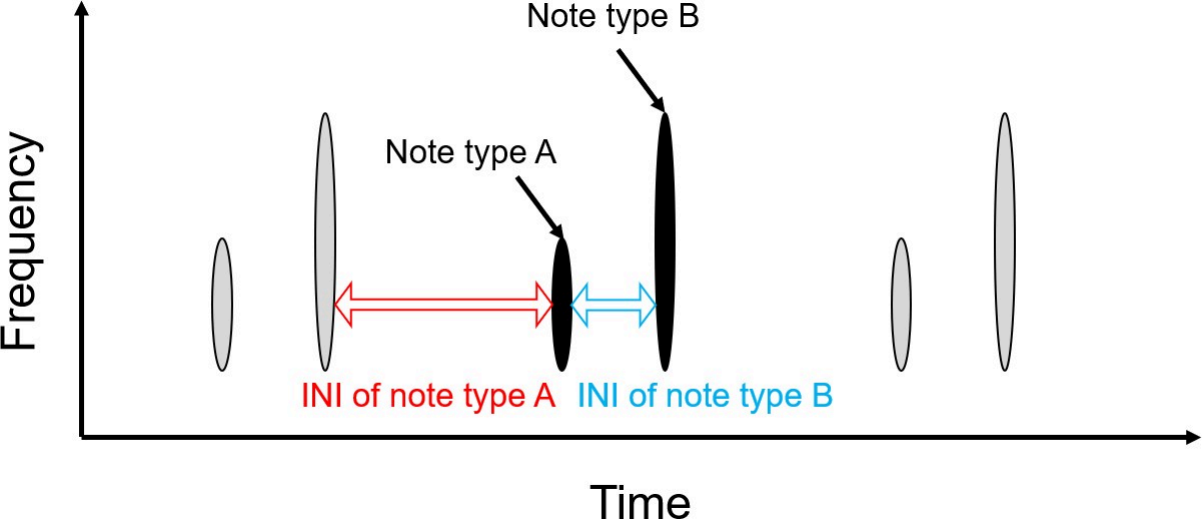
Illustration of Inter-Note Intervals (INIs) measured in both datasets (Cook Strait and Gisborne). INIs measured between each detected fin whale ‘doublet’ calls (red) and between the two notes of the ‘doublet’ calls (blue).

In both the Gisborne and Cook Strait datasets, spectrograms of the 10.wav file containing ‘doublet’ calls were visually inspected for the presence of High Frequency (HF) components. The HF components are naming conventions for the song types which allow the comparison to other song types from other studies. The frequency of each HF component was assessed by measuring the peaks in the spectrogram (PSD) between 60 Hz and 100 Hz (Fig 3), a bandwidth known to contain the HF components of the fin whale ‘doublet’ calls [8, 21, 22]. The peak detection occurred when the PSD reached at least 90 dB for the Cook Strait dataset and -50 relative dB for the Gisborne dataset.

As soon as the detector for the ‘doublet’ calls detection was validated, no change in the detector was made for detecting the HF components and the INI. To perform a manual validation of the detector, 250.wav files were randomly chosen from the outputs of each station to determine the detector performance on the real dataset. These 250.wav files corresponded to ~1.3% of the files from each AMAR deployed in Cook Strait and ~63% of the files from each seismometer deployed off Gisborne. Despite representing different proportions of each dataset, the same number of files was selected for consistency. Details of how the performance was assessed and the obtained results can be found in S1 Appendix.

### Inter-Note Interval of ‘doublet’ calls

The Inter-Note Interval INI [24, 31], or the time interval between fin whales ‘doublet’ calls, was measured in the time domain of the pressure time-series using MATLAB and averaged between each detected ‘doublet’ calls per month (INI of note type A, Fig 3) and between the two calls of each ‘doublet’ calls (INI of note type B, Fig 3). The INIs are naming conventions for the song types which allow the comparison to other song types from other studies.

When ‘doublet’ calls were detected, INI was calculated between ‘doublet’ calls (red) and between the two notes composing each ‘doublet’ calls (blue) (Fig 3). The INI of each ‘doublet’ calls was calculated by measuring the time interval between the centre of note type A of a ‘doublet’ calls and the centre of note type B of the previous ‘doublet’ calls (as described in Castellote et al., 2011 [10]). The INI between note type A and note type B composing each ‘doublet’ calls was calculated by measuring the time between the centres of the two notes (as described in Castellote et al., 2011 [10]).

## Results

### Occurrence over time and peak frequency of ‘doublet’ calls

**Fig 4:**
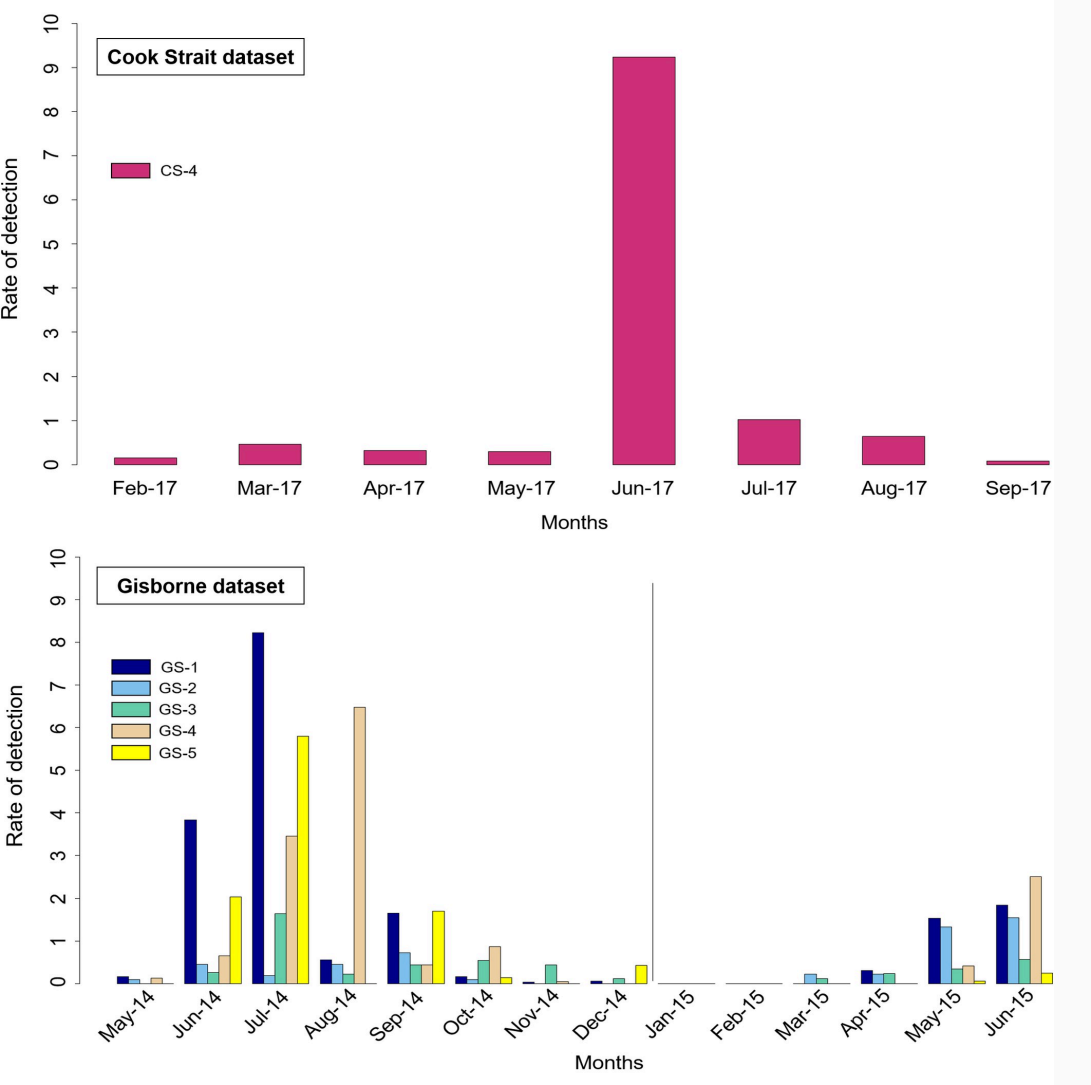
Abundance ratio of ‘doublet’ calls for each month per year distributed in the whole year. Upper: ‘Doublet’ calls detected in the Cook Strait dataset during 2017. ‘Doublet’ calls were only detected at CS-4 on the east side of Cook Strait. Lower: ‘Doublet’ calls in the Gisborne dataset, on the east side of New Zealand, during 2014–2015.

Fin whale ‘doublet’ calls occurred most often during austral winter (Fig 4). Of the four stations in Cook Strait, ‘doublet’ calls were only detected on CS-4 located on the east side of the Cook Strait.

The ‘doublet’ calls were mainly detected in June 2017 in the east side of the Cook Strait (CS-4) and in June 2014 (GS-1), July 2014 (GS-1, GS-4, and GS-5) and August 2014 (GS-4) at the seismometer station offshore Gisborne. No ‘doublet’ calls were detected in 2016 in the east side of the Cook Strait.

Cook Strait and Gisborne datasets showed similar peak frequency average values for each notes (type A and B) composing every detected ‘doublet’ calls (Table 2): ~20.3 Hz for note type A and ~22.2 Hz for note type B.

**Table 2:** Total number of note types detected, mean peak frequency and standard deviation for each dataset.

		Total number of fin whales ‘doublet’ calls detected	Timing of first detection—timing of last detection (DD/MM/YYYY)	Note type A		Note type B	
				Mean peak frequency (Hz)	Standard deviation	Mean peak frequency (Hz)	Standard deviation
Cook Strait	CS-4	529	15/02/2017–05/09/2017	20.1	1.04	22.4	0.81
Offshore Gisborne	GS-1	793	27/05/2014–16/06/2015	20.6	1.64	21.8	0.79
	GS-2	229	17/05/2014–19/06/2015	20.3	0.64	22.2	1.16
	GS-3	211	14/06/2014–15/06/2015	19.9	0.94	22.5	0.83
	GS-4	647	25/05/2014–18/06/2015	20.6	0.67	22.0	1.25
	GS-5	449	19/06/2014–17/06/2015	20.2	0.83	22.3	1.22

Cook Strait dataset, east side, during 2017 (CS-4) and Gisborne dataset, offshore Gisborne, during 2014–2015.

No significant seasonal and spatial variation of the peak frequency of each note was observed (Kruskal-Wallis test of note type A and B, χ^2^ = 5, df = 5, p-value = 0.4159).

### High frequency components and Inter-Note Interval of ‘doublet’ calls

**Fig 5:**
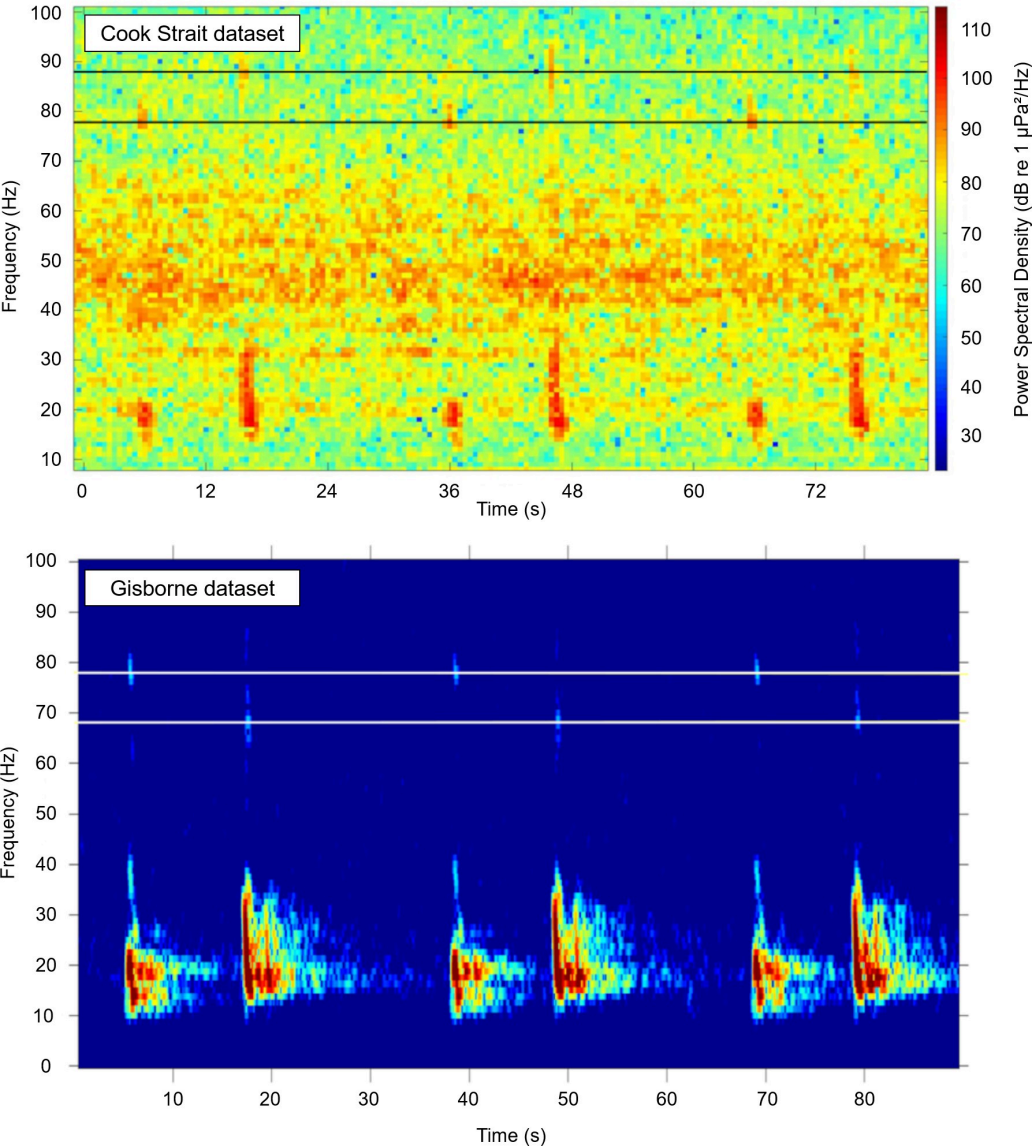
Dual HF components (lines added to denote upper frequencies). Upper: Cook Strait dataset, east side, during 2017 (CS-4), shows HF of 78 Hz and 88 Hz. No detections occurred on the west side. Lower: Gisborne dataset, offshore Gisborne, during 2014–2015, shows HF of 78 Hz and 68 Hz.

The HF components frequencies are ~80 Hz and ~90 Hz for the Cook Strait dataset and ~70 Hz and ~80 Hz for the Gisborne dataset (Fig 5 and Table 3).

**Table 3:** Number of HF components analyzed, mean HF peak frequency and standard deviation for each dataset.

		Number of HF components analyzed	1^st^ component		2^nd^ component	
			Mean peak frequency (Hz)	Standard deviation	Mean peak frequency (Hz)	Standard deviation
Cook Strait	CS-4	232	77.6	1.36	88.2	2.32
Offshore Gisborne	GS-1	204	78.4	1.85	67.8	1.72
	GS-2	41	77.6	2.73	66.4	2.15
	GS-3	12	76.8	2.32	67.0	3.03
	GS-4	174	76.0	2.45	67.4	1.85
	GS-5	196	77.8	2.14	68.8	1.60

Cook Strait dataset, east side, during 2017 (CS-4) and Gisborne dataset, offshore Gisborne, during 2014–2015.

The HF components do not appear to change over time within each dataset. However, the HF components vary between Cook Strait and Gisborne datasets. The mean frequency of the 1^st^ HF component of the Cook Strait dataset is lower than the mean frequency of the 1^st^ HF component of the Gisborne dataset. Conversely, the mean frequency of the 2^nd^ HF component is higher for the Cook Strait dataset than the mean frequency of the 2^nd^ HF component of the Gisborne dataset (Fig 5 and Table 3). This means that there is a difference in alternation of both HF components between the two areas. Therefore, these HF components are not simply harmonics of the ‘doublet’ calls that could have been lost due to distance.

**Fig 6:**
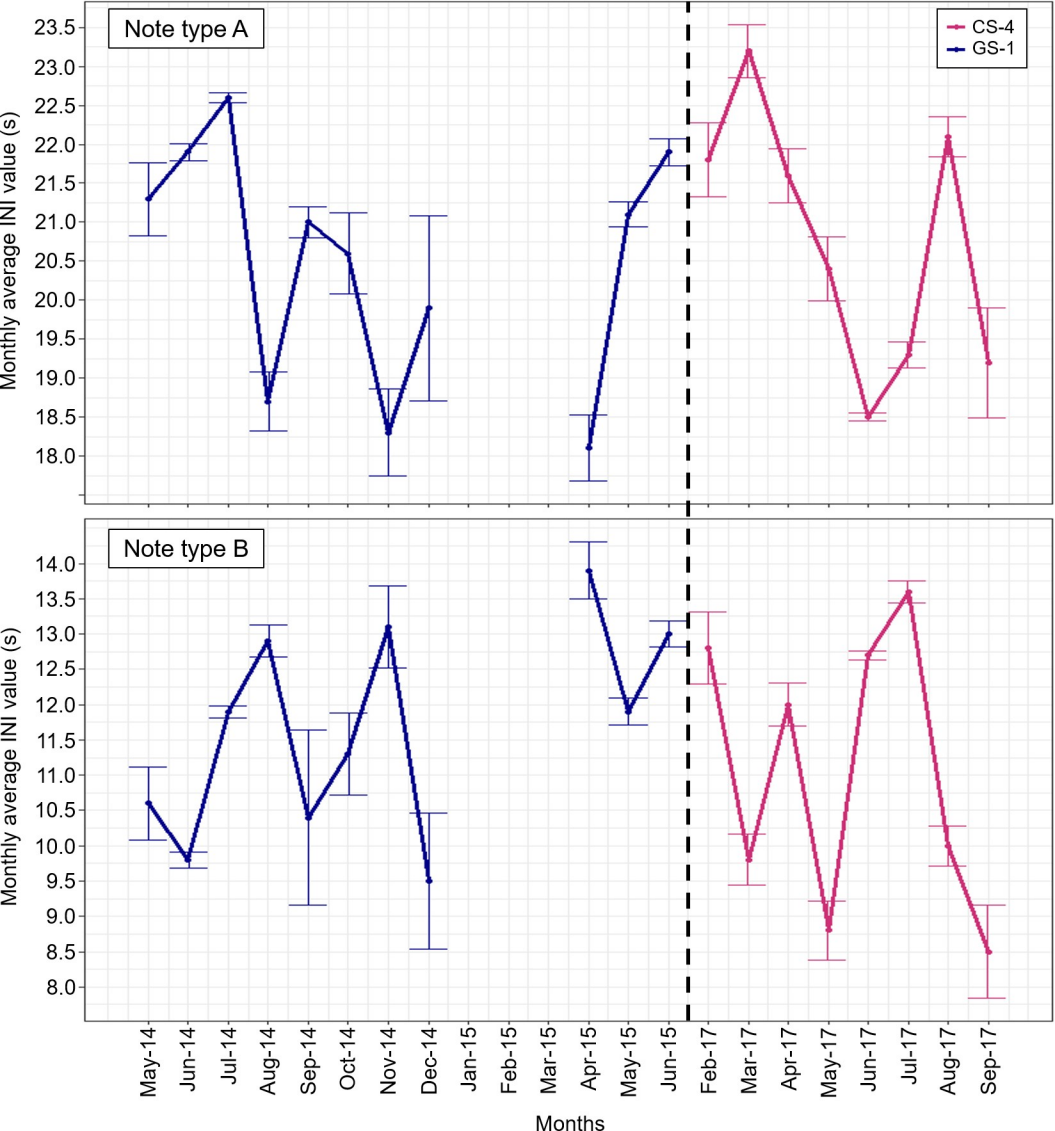
Monthly average values of INI for the two note types (A and B) for both datasets. Cook Strait (2017, CS-4 east side) and Gisborne (2014 and 2015; here: only GS-1 is shown) datasets. Error bars: standard error of the mean.

The monthly average INI values have been calculated for the two note types A and B (Fig 6). The INI of note type A and note type B are contained in a similar interval for both datasets: between 18 and 23 s for note type A and between 8 and 13 s for note type B approximately. However, there are some variations of INI of about 2 s within each dataset. Some of these variations may be uncertain (Fig 6 error bars) especially in December 2014, September 2017 for both note types and December 2014 for note type A. The INI of note type A drops in August and November 2014 and increases in May 2015 for GS-1. It also drops continuously from April to June 2017 before rising in August 2017 and dropping the next month for CS-4. The INI of note type B increases in July 2014 and drops in September 2014 and December 2014 for GS1. It drops in March and May 2017 and drops continuously from August to September 2017 for CS-4.

Both datasets showed similar total INI average (Table 4): ~20.7 s for that preceding note type A and ~10.5 s for that preceding note type B.

**Table 4:** Total number of note types detected, mean INI and standard deviation for each dataset.

		Total number of fin whales ‘doublet’ calls detected	Timing of first detection—timing of last detection (DD/MM/YYYY)	Note type A		Note type B	
				Mean INI (s)	Standard deviation	Mean INI (s)	Standard deviation
Cook Strait	CS-4	529	15/02/2017–05/09/2017	20.8	1.66	11.0	1.98
Offshore Gisborne	GS-1	793	27/05/2014–16/06/2015	20.5	1.54	11.5	1.74
	GS-2	229	17/05/2014–19/06/2015	20.1	1.96	10.8	1.60
	GS-3	211	14/06/2014–15/06/2015	21.3	1.98	10.5	2.01
	GS-4	647	25/05/2014–18/06/2015	20.9	1.34	10.2	1.49
	GS-5	449	19/06/2014–17/06/2015	20.2	1.46	10.3	1.78

Cook Strait dataset, east side, during 2017 (CS-4) and Gisborne dataset, offshore Gisborne, during 2014–2015.

The INI does not seem to change significantly over time within and between each dataset.

Furthermore, there was no seasonal variation of INI in each station and there was no statistical variation between all INI of both datasets (α = 0.05) (Kruskal-Wallis test for INI of note type A and B, χ^2^ = 5, df = 5, p-value = 0.4159).

The song types (HF components and INI) described in this study were compared with previous studies on fin whales song types (Table 5).

**Table 5:** Examples of fin whales song types described in the literature.

Location	Longitude	Years	INI (s)	HF (Hz)	Reference
New Zealand	174-176E and 178.5-179E	2016–17 and 2014–15	~20.7, ~10.5	~80, ~90 and ~70, ~80	This study
East Antarctica	65E	2003–4	~14	99	Širović et al., 2009
West Antarctic Peninsula	50-65W	2003–4	~14	89	Širović et al., 2009
Southern Kerguelen Plateau	81E and 75E	2005	~17, ~8	99	Gedamke, 2009
Tasmania	145E	2006–7	~18, ~10	82, 94	Gedamke, 2009
West Australia	115E	2004–7	~27, ~13	99	Gedamke, 2009
Dumont d’Urville	141E	2007	~10, ~8	82, 94	Gedamke, 2009
Juan Fernandez	79W	2016	~14	85.3	Buchan et al., 2019
South Australia	115E,141E and 153E	2009–17	~28, ~15; ~33, ~17; ~17, ~9	NA	Aulich et al., 2019
Great Barrier Island, New Zealand	176E	1997	17, 9	NA	McDonald, 2006

## Discussion

Fin whale ‘doublet’ calls were detected predominantly during the austral winter on the east side of Cook Strait and offshore Gisborne. A previous study also detected fin whales off Great Barrier Island in New Zealand waters during winter, from June to September [7]. Only male fin whales sing [14] so the presence of fin whales in these area during the austral winter could be explained as breeding purpose [4, 14]. The Cook Strait and Gisborne datasets did not overlap in time, therefore limiting the spatial coverage of this study. Additionally, it was not possible to evaluate the number of singing animals present in each area.

The occurrence of fin whales ‘doublet’ calls in New Zealand waters is not consistent across years. On the east side of the Cook Strait, ‘doublet’ calls were detected in 2017 but not in the previous year. Similarly, more ‘doublet’ calls were detected off Gisborne in 2014 than 2015. Despite the close distance between GS-1 and GS-2, there was a difference in detection between these two seismometers. Being seismometers instruments that sit on the seafloor, it is possible that directional blockage due to bathymetric features may be giving each of the instrument different detection capability. The absence of detections on the west side of Cook Strait may indicate habitat preference. However, acoustic monitoring needs to occur in more areas off the West Coast of New Zealand to confirm this hypothesis. McDonald (2006) [7] highlighted the presence of fin whales east of New Zealand. The preference for the eastern offshore area may be due to the current system of this region. The East Auckland Current goes south through the East Cape Current to the Cook Strait [37]. There, the current undergoes a retroflexion with the Wairarapa Eddy and continues towards the east to close the South Pacific Gyre [37]. Furthermore, after the retroflexion with the Wairarapa Eddy, the current approaches the Southland Current which is partly composed of Subantarctic Water [38] and linked to fronts of the Antarctica [39]. Some fin whales in the North Pacific Ocean are known to take advantage of the California Current for migration or food for resident populations [40], thus fin whales around New Zealand may use currents off the East Coast as corridors during their migration to reach more southerly latitudes in the summer. Further research is needed to assess these hypotheses in New Zealand. Fin whales may also take advantage of the productivity of these currents to find prey and male fin whales may attract females where these prey aggregate [14].

The peak frequencies of type A notes, type B notes and HF components, as well as the INIs, of the New Zealand fin whales ‘doublet’ calls were measured. The peak frequencies of HF components appeared to be constant over time within but not between datasets. In the Cook Strait area, the 1^st^ HF component of the ‘doublet’ calls had a lower peak frequency than the 2^nd^ HF component, whereas offshore Gisborne the 1^st^ HF component of the ‘doublet’ calls had a higher peak frequency than the 2^nd^ HF component. The peak frequencies of type A and B notes as well as the total INI average seem to remain constant within each dataset and between all datasets of both locations (Cook Strait and offshore Gisborne). However, the monthly INI average shows some variations (about 2 s) within each dataset. Considering the uncertainty as well as the fact that ‘doublet’ calls were mostly found in a short time interval (one month or two consecutives), these variations could be due to an odd singer. Longer datasets with ‘doublet’ calls that spread more over time would have helped to see if these variations were only isolated or if a trend could be noticed. Consequently, there is no evidence of the song types changing over time, like those described in Oleson et al. 2014 [29] or Weirathmueller et al. 2017 [31].

The song types described in our study were compared with song types previously described in the literature (Table 5). The INI values from our study (~20.7 and ~10.5 s) are similar to those from Gedamke (2009) [8] in Tasmania (~18 and ~10 s) and the southern Kerguelen Plateau (~17 and ~8 s). The INI values of one of the songs studied in the South of Australia by Aulich et al. (2019) [26] (~17 and ~9 s) as well as those from Great Barrier Island in McDonald (2006) [7] (17 and 9 s) also match closely the INI values from our study. The slight difference between the values in our study compared to the other studies cited above [7, 8, 26] may be explained by the time interval between the data collection and a manual approximation method (the INI values in the paper of Gedamke (2009) [8] and Aulich et al. (2019) [26] were manually estimated from the spectrograms provided). The HF components frequencies from our study in the Cook Strait area (~80 Hz and ~90 Hz) are similar to those from Gedamke (2009) [8] in Tasmania and Dumont d’Urville (82 Hz and 94 Hz). Similarly, the difference between the HF components frequencies in our study and those from Gedamke (2009) [8] may be explained by the time interval between the data collection. In fact, there is a well-established tendency of baleen whales (including fin whale) call frequency to drop over time in the southern hemisphere [41, 42]. The HF components frequencies from our study offshore Gisborne (~70 Hz and ~80 Hz) partly match the INI values from Gedamke (2009) [8] (~82 Hz and ~94 Hz) and Buchan et al. (2019) [21] (85.3 Hz). Therefore, it appears that song types described in our study may be related to the song types recorded between Tasmania and Antarctica and described by Gedamke (2009) [8] (at least for the Cook Strait dataset).

Our findings suggest that some fin whales wintering in New Zealand waters may be part of a broader ‘acoustic population’ whose range extends west to southern Australia and south to Antarctica. Fin whales appear to occur in New Zealand more than previously recognized and are currently listed as ‘migrant’ and ‘endangered’ by the New Zealand Threat Classification system, according to sighting records and using museum specimens [43]. Lack of data on fin whales abundance and distribution around New Zealand is likely confounded by their preference for offshore waters [5], in areas that can be costly and logistically challenging to survey.

The detector developed in this study was designed to only detect songs composed of the stereotyped pattern of ‘doublet’ calls [23]. Therefore, this template-based detector was restrictive but selective to be sure that the targets detected were fin whales [23]. Although ‘doublet’ calls are the predominant fin whale song type within the latitudes of our study [23], our results suggest that a review of southern hemisphere fin wale song types should be further explored to confirm the existence of one or more ‘acoustic populations’ which the New Zealand one is part of.

## Supporting information

S1 Appendix Validation of the detector with the receiver operating characteristics curves and estimation of the detector performance. (DOCX)

S1 Table Confusion matrix that shows the four possible outputs for the comparison between detection and reality. (TIF)

S2 Table Fin whale detector training trials for Cook Strait training dataset. (TIF)

S3 Table Fin whale detector training trials for Gisborne training dataset. (TIF)

S4 Table Confusion matrix of the fin whale detector on the Cook Strait dataset, east side, during 2017. (TIF)

S5 Table Performance of the fin whale detector on the Cook Strait dataset, east side, during 2017. (TIF)

S6 Table Confusion matrices of the fin whale detector on the Gisborne dataset, offshore Gisborne, during 2014–2015. (TIF)

S7 Table Performance of the fin whale detector on the Gisborne dataset, offshore Gisborne, during 2014–2015. (TIF)

S1 Data Dataset to replicate this study findings. (XLSX)

S1 Raw images Raw images used for manuscript and S1 Appendix. (PDF)
