# Supplementary material for: Fin whale acoustic populations present in New Zealand waters: Description of song types, occurrence and seasonality using passive acoustic monitoring
Source: PLoS One. 2021 Jul 14;16(7):e0253737. doi: 10.1371/journal.pone.0253737 (PMC8279366; doi:10.1371/journal.pone.0253737)
Supplement: S1 Appendix — (DOCX) [file pone.0253737.s001.docx]

**Supporting Methods**

Alexandra N. Constaratas, Mark A. McDonald, Kimberly T. Goetz, Giacomo Giorli

**Validation of the detector with the Receiver Operating Characteristics curves and estimation of the detector performance**

Receiver Operating Characteristics (ROC) curves were used to optimize the threshold and binarization parameters used by the fin whale detector. Two training datasets (one for the Cook Strait AMAR dataset, and one for the Gisborne seismometer dataset) consisting of 68 .wav files were created. These 68 .wav files correspond to 0.1 % of the whole Cook Strait AMAR dataset and 3.4 % of the whole Gisborne seismometer dataset. The 68 .wav files were selected manually after a visual check of their spectrograms to be sure of their content. Both the training datasets contained 34 .wav files with the target signal (i.e. fin whale ‘doublet’ calls), and 34 .wav files with no target signal. After running the detector on these training datasets, four outputs were possible (Table 1): True Positive (TP; detected and present), False Negative (FN; not detected but present), False Positive (FP; detected but absent) and True Negative (TN; not detected and absent).

**Table 1. Confusion matrix that shows the four possible outputs for the comparison between detection and reality.**


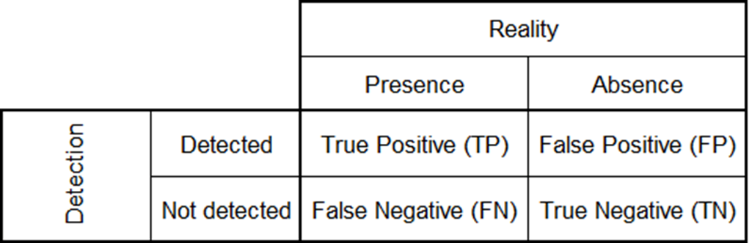


The ROC is based on the determination of TP and FP ratios. A good detector is characterized by high TP ratio and small FP ratio. If the TP and FP ratios are optimized, the TN and FN ratios are optimized as well (TP + FN = 100 % and TN + FP = 100 %). The detector was run 10 times on each training dataset to make 10 trials per dataset. In each trial, the two parameters of the fin whale detector were changed: binarization threshold and cross-correlation threshold. The combination of parameters that yielded the best detector performance (i.e. the best balance between TP and FP ratios) for each training dataset was saved and used for the detection in the real datasets.

After the 10 trials on each training dataset (68 .wav files), a ROC curve was obtained for the fin whale detector for each dataset. TP, FP, FN and TN ratios were given by the number of ‘doublet’ calls detected/not detected in all the 68 .wav files divided by the total number of ‘doublet’ calls of all the 68 .wav files. The values of the parameters that returned the best classification performance were selected to be used on the real datasets. The best detector performance was obtained using a binarization threshold of 88 dB and a cross-correlation threshold of 0.5 for the Cook Strait training dataset (Table 2). A binarization threshold of -100 relative dB and a cross-correlation threshold of 0.5 gave the best performance of the detector for the Gisborne training dataset (Table 3).

**Table 2. Fin whale detector training trials for Cook Strait training dataset.**

Red: Best performance trial. Values of parameters of Trial 4: binarization threshold = 88 dB and cross-correlation threshold = 0.5).


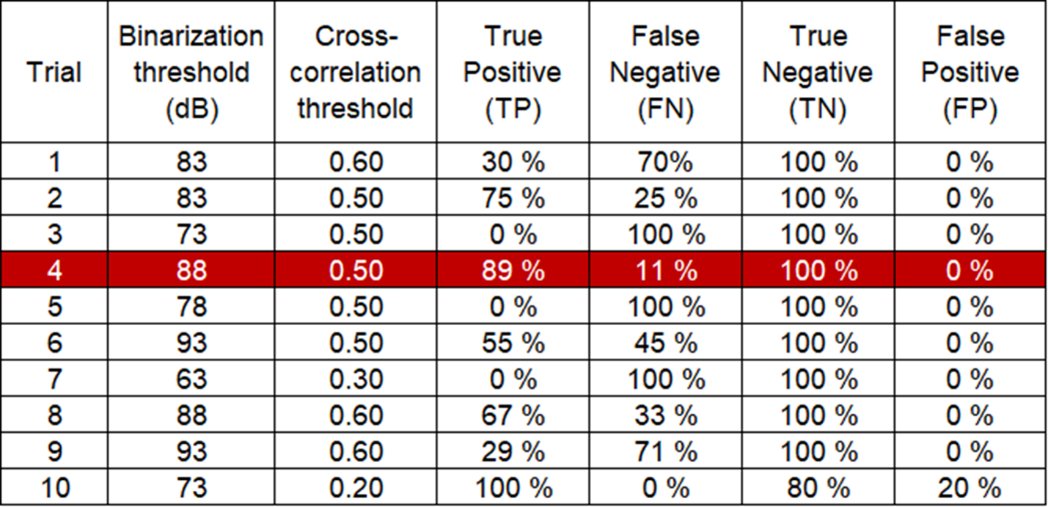


**Table 3. Fin whale detector training trials for Gisborne training dataset.**

Red: Best performance trial. Values of parameters of Trial 3: binarization threshold = -100 relative dB and cross-correlation threshold = 0.5).


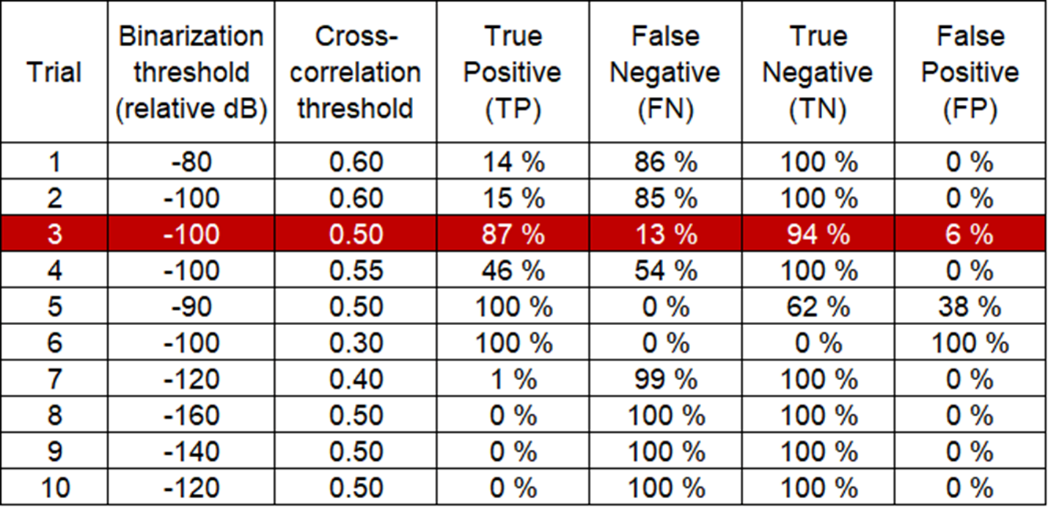


The thresholds from Trial 4 (Table 2) were used to run the detector on the Cook Strait (east and west) dataset and the thresholds from Trial 3 (Table 3) were used to run the detector on the Gisborne dataset.

After launching the detector on all the different stations, 250 .wav files were randomly selected from all the stations to determine the detector performance. The spectrograms of these .wav files were visually checked to see if they could represent as many various environmental conditions as possible: background noise present and absent, high and low signal-to-noise ratio notes. The detector was therefore designed to capture as many calls as possible in most conditions. The performance of the detector was assessed computing four parameters: precision (P), recall (R), F-score (F) and accuracy (A). P, R, F and A were calculated using the following equations:

$$P=\frac{TP}{TP+FP} R=\frac{TP}{TP+FN} F=\frac{\left( 1+\beta^{2} \right)P*R}{\beta^{2}P+R} A=\frac{TP+TN}{N}$$

where TP, FN, TN, FP and N are respectively the True Positive, False Negative, True Negative and False Positive ratios, and the number of .wav files analyzed (250 in this case).

P represents the proportion of detections that are TP. As an example, a P value of 0.9 means that 90% of the detections were correct without saying whether all ‘doublet’ calls in the dataset were identified. R represents the proportion of fin whale ‘doublet’ calls in the dataset that are detected by the detector. An R value of 0.8 means that 80% of all fin whale ‘doublet’ calls in the dataset were detected without saying how many detections were incorrect. Thus, a perfect detector would have P and R values equal to 1. The F-score is a combined measure of P and R. An F-score of 1 indicates perfect performance of the detector. β is the relative weight between the recall and the precision. For example, a β of 0.5 means the recall has half the weight of the precision. Finally, A represents how likely the detectors identify TP or TN. If A is higher than 0.5, then the detector was better at identifying TP or TN than FP or FN.

The validation of the detection was done manually: the spectrogram of each .wav file was visually inspected to check whether the classification was correct or not, to calculate the TP, FP, TN, and FN ratios as well as the performance parameters (Table 4 and 5 for the Cook Strait dataset; Table 6 and 7 for the Gisborne dataset).

Relative abundance of fin whale ‘doublet’ calls in the Cook Strait and offshore Gisborne over time was computed as a ratio between the number of ‘doublet’ calls detected in each month divided by the number of minutes recorded per month (i.e. effort). This normalization enabled to compare the results between datasets.

**Table 4. Confusion matrix of the fin whale detector on the Cook Strait dataset, east side, during 2017.**

CS: Cook Strait, TP: True Positive, FN: False Negative, FP: False Positive, TN: True Negative.


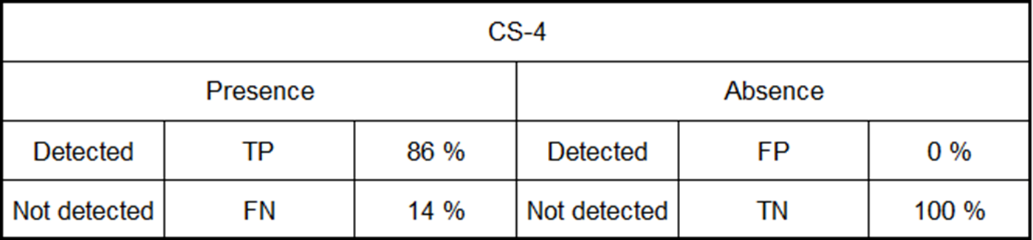


No fin whales were detected in the west side of the Cook Strait, either in 2016 or 2017. The detector returned very few FP detections. Moreover, when there was no detection, there was no fin whale (yielding a TN of 100 %). No detections also resulted for the east side of the Cook Strait in 2016. Fin whales were only detected in the east side of the Cook Strait in 2017 (i.e. station CS-4 of the east side).

**Table 5. Performance of the fin whale detector on the Cook Strait dataset, east side, during 2017.**

CS: Cook Strait.


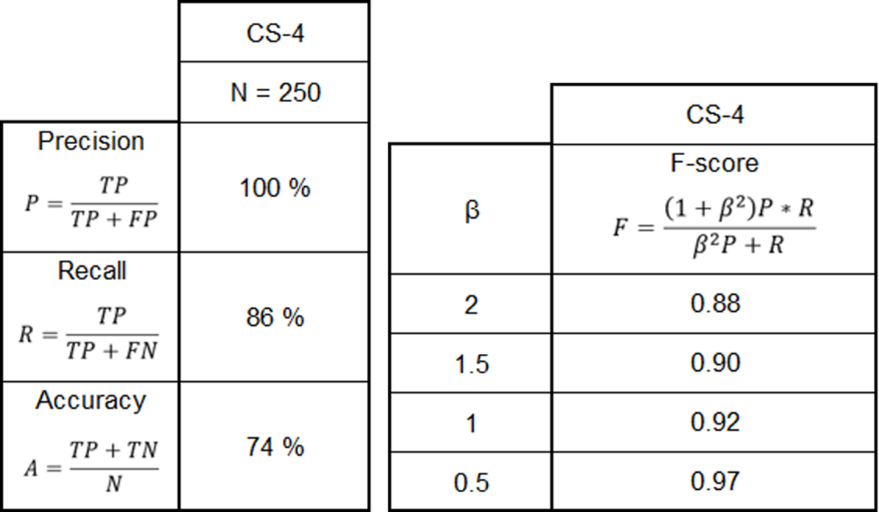


**Table 6. Confusion matrices of the fin whale detector on the Gisborne dataset, offshore Gisborne, during 2014-2015.**

GS: Gisborne, TP: True Positive, FN: False Negative, FP: False Positive, TN: True Negative.


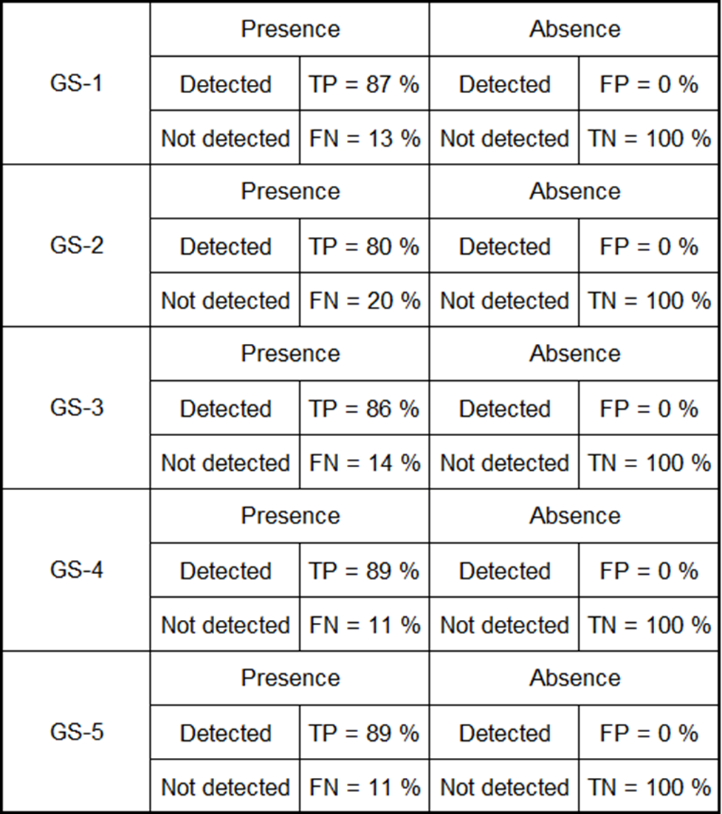


Fin whales were detected in every station of the Gisborne dataset. Moreover, when there was no detection, there was no fin whale (yielding a TN of 100 %).

**Table 7. Performance of the fin whale detector on the Gisborne dataset, offshore Gisborne, during 2014-2015.**

GS: Gisborne, N: number of files, P: precision, R: recall, A: accuracy.


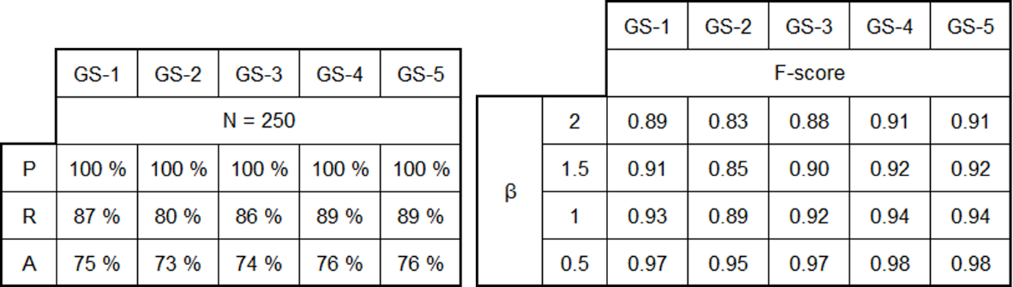


The fin whale detector performed the best for station GS-4 and station GS-5, with a TP ratio of 89 %, and the worst for station GS-2 with a TP ratio of 80 %. The fin whale detector accuracy was greater than 50 % (74 % for the Cook Strait dataset, more than 73 % for the Gisborne dataset).
