# Supplementary figures and images for: Fin whale acoustic populations present in New Zealand waters: Description of song types, occurrence and seasonality using passive acoustic monitoring
Source: PLoS One. 2021 Jul 14;16(7):e0253737. doi: 10.1371/journal.pone.0253737 (PMC8279366; doi:10.1371/journal.pone.0253737)

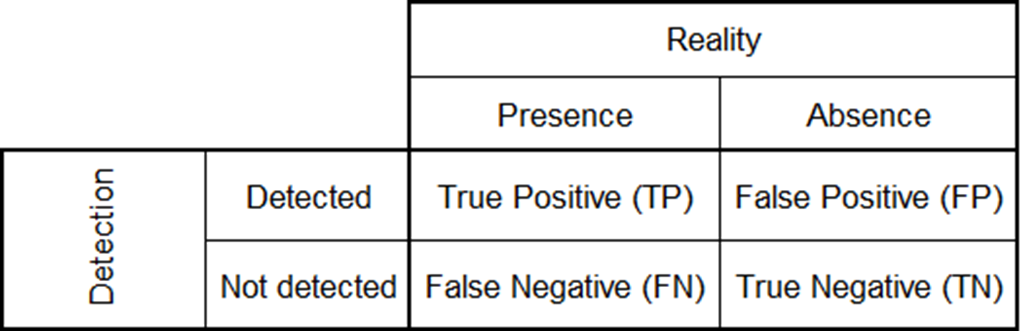

Supplement: S1 Table — (TIF) [file pone.0253737.s002.tif]

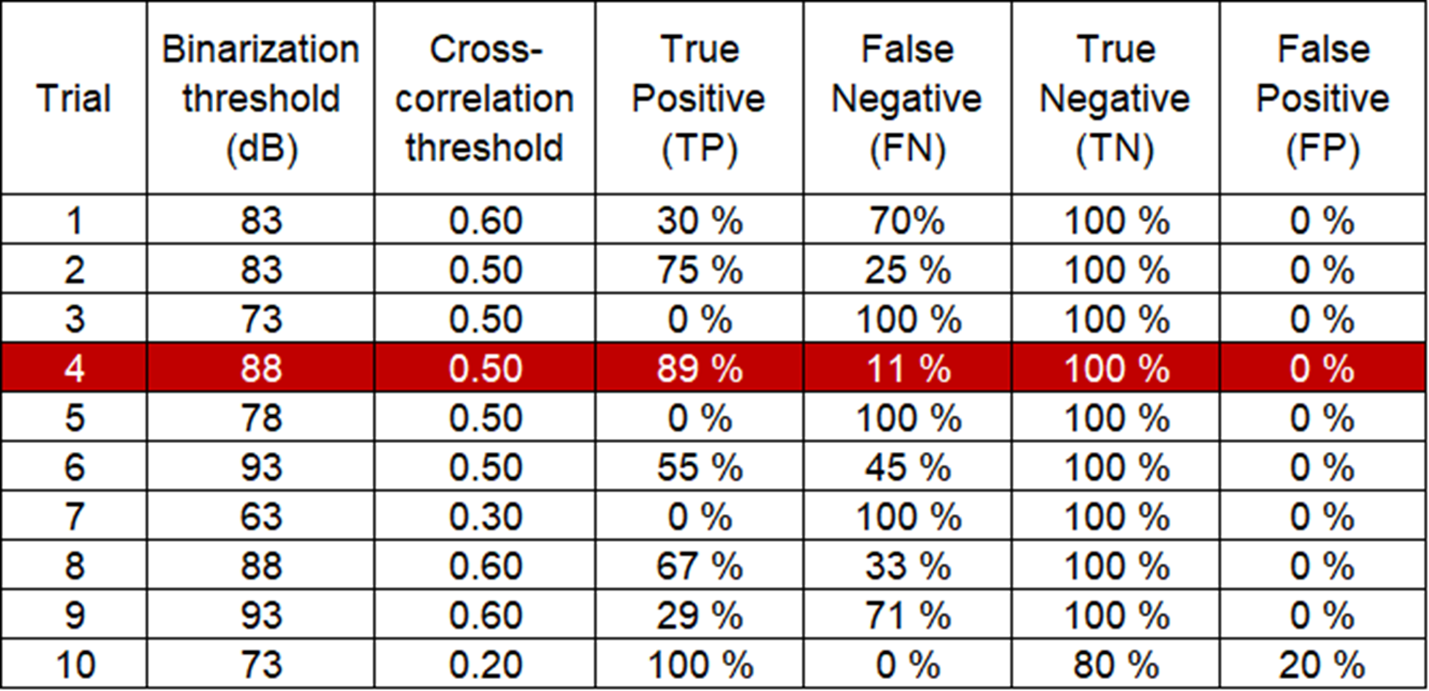

Supplement: S2 Table — (TIF) [file pone.0253737.s003.tif]

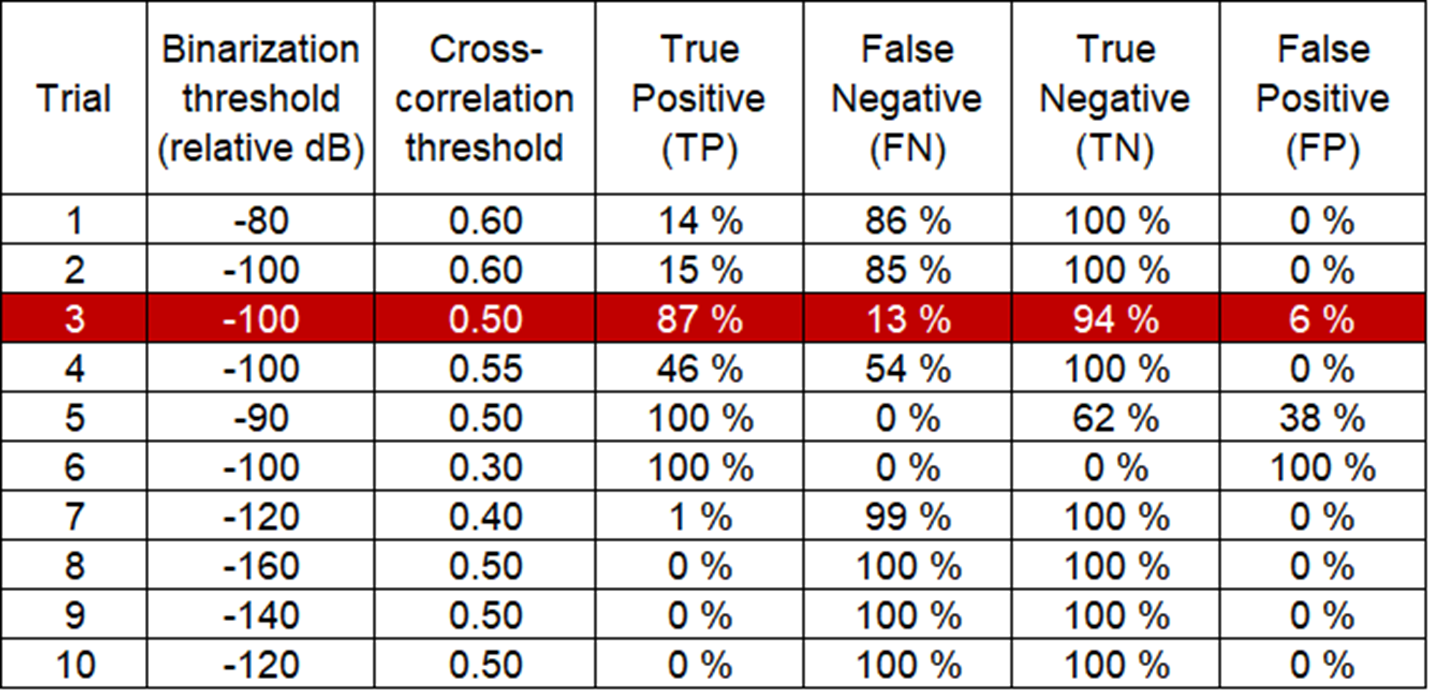

Supplement: S3 Table — (TIF) [file pone.0253737.s004.tif]

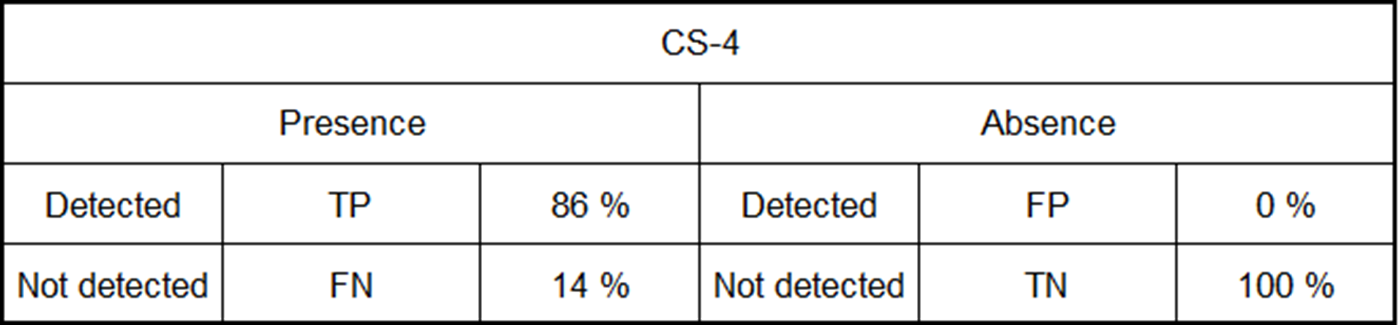

Supplement: S4 Table — (TIF) [file pone.0253737.s005.tif]

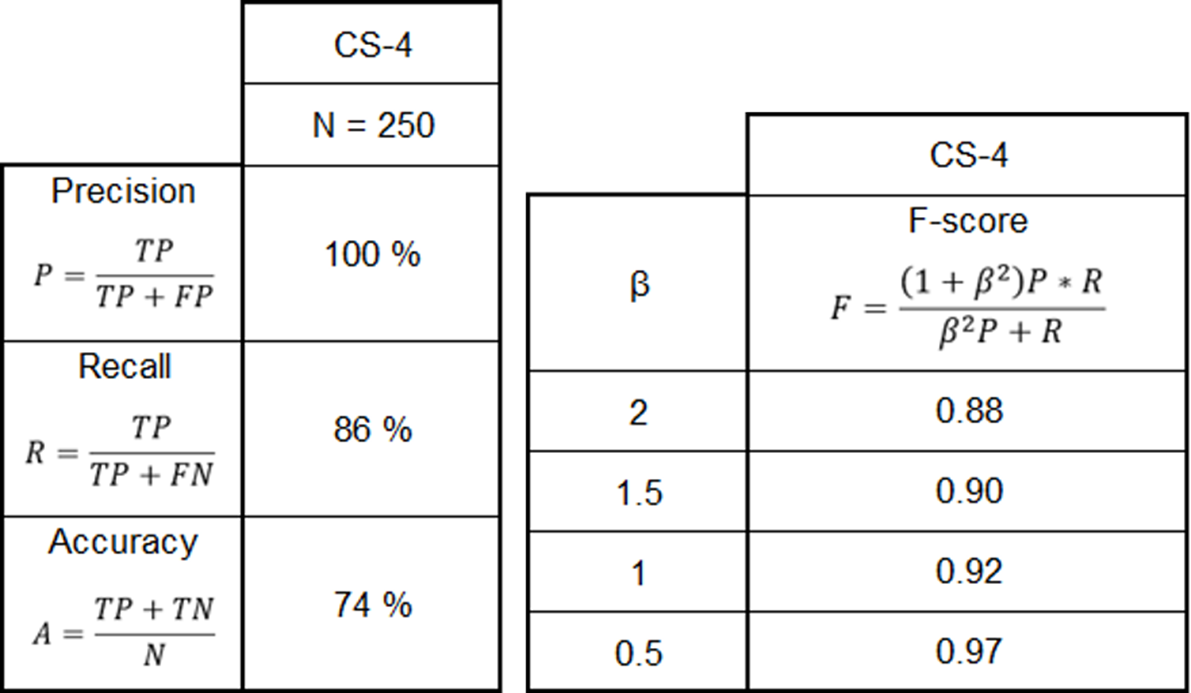

Supplement: S5 Table — (TIF) [file pone.0253737.s006.tif]

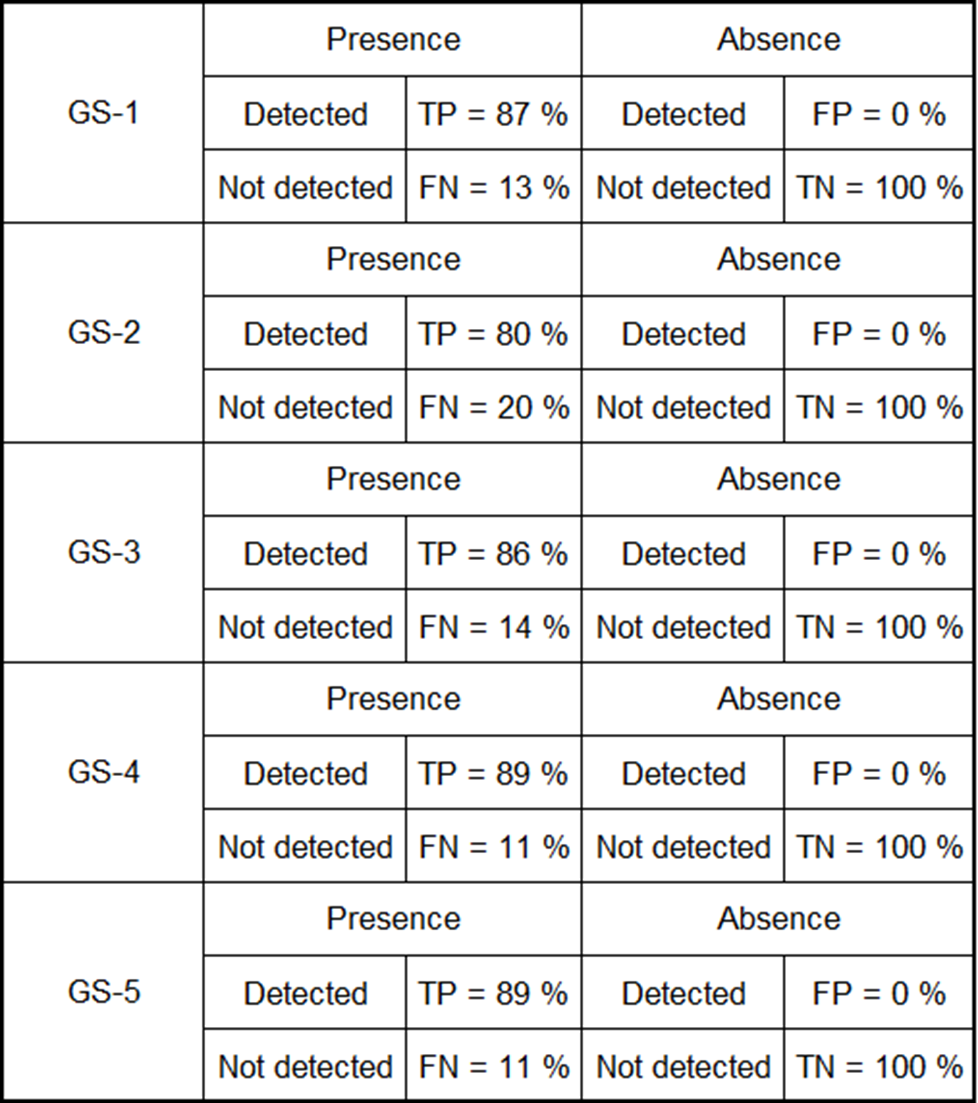

Supplement: S6 Table — (TIF) [file pone.0253737.s007.tif]

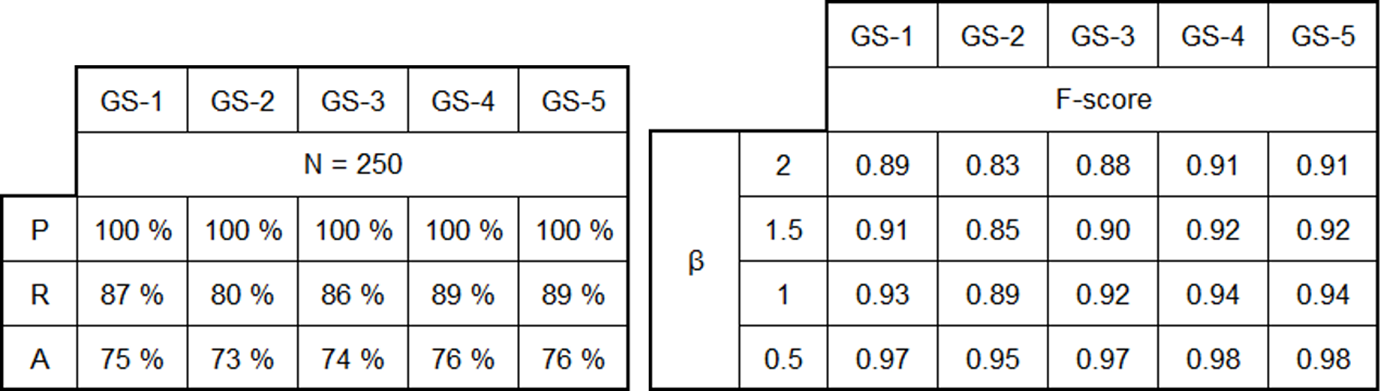

Supplement: S7 Table — (TIF) [file pone.0253737.s008.tif]
