# Supplementary material for: Fin whale acoustic populations present in New Zealand waters: Description of song types, occurrence and seasonality using passive acoustic monitoring
Source: PLoS One. 2021 Jul 14;16(7):e0253737. doi: 10.1371/journal.pone.0253737 (PMC8279366; doi:10.1371/journal.pone.0253737)
Supplement: S1 Raw images — (PDF) [file pone.0253737.s010.pdf]

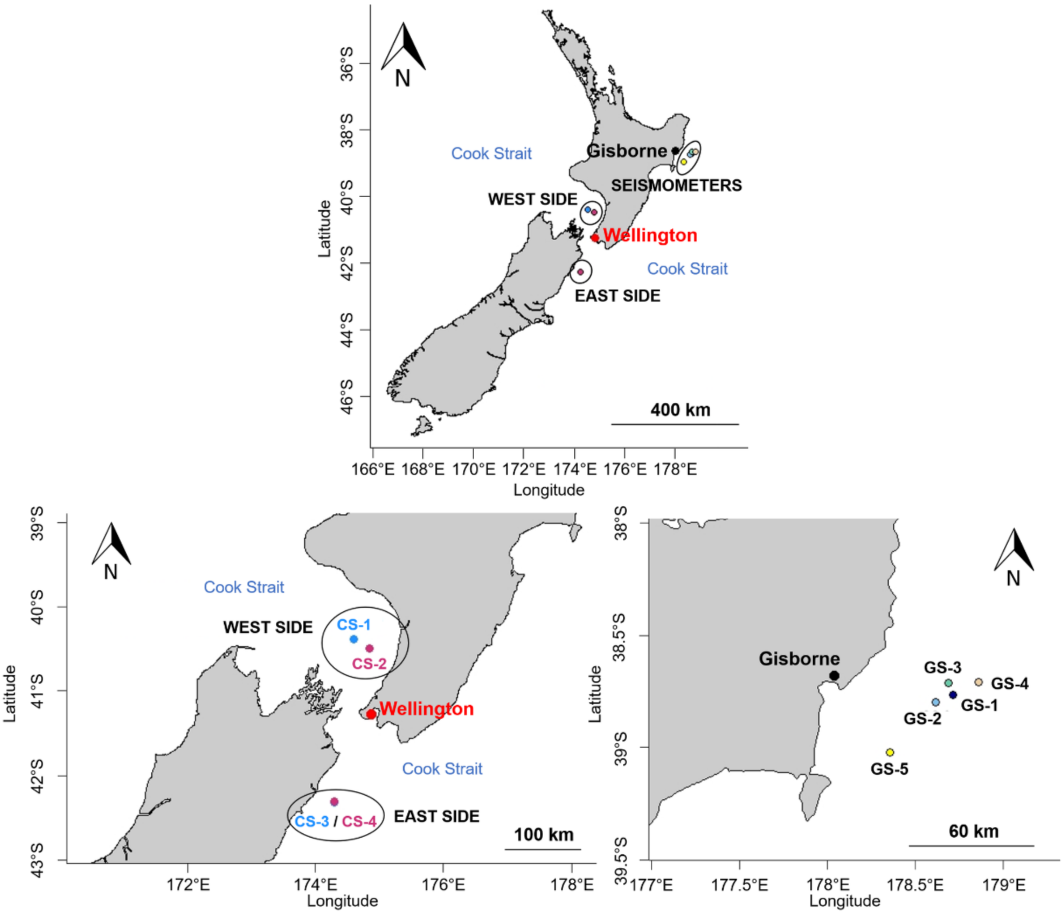

Fig 1

	Side	Station	Instrument	Latitude	Longitude	Depth (m)	Start date of recording (DD/MM/YYYY)	End date of recording (DD/MM/YYYY)	Number of files analyzed	Total duration recording (days)
Cook Strait	West	CS-1	AMAR	-40.4195	174.5074	110	04/06/2016	21/12/2016	19 040	139
		CS-2	AMAR	-40.5259	174.7571	100	15/02/2017	04/09/2017	17 982	131
	East	CS-3	AMAR	-42.3087	174.2145	1 251	28/04/2016	21/12/2016	19 022	138
		CS-4	AMAR	-42.3071	174.2139	1 200	15/02/2017	08/09/2017	19 092	139
Gisborne	East	GS-1	Seismometer	-38.7459	178.6789	995	13/05/2014	20/06/2015	404	404
		GS-2	Seismometer	-38.7771	178.5835	930	11/05/2014	20/06/2015	406	406
		GS-3	Seismometer	-38.6946	178.6506	1 023	11/05/2014	21/06/2015	405	405
		GS-4	Seismometer	-38.6888	178.8199	1 712	13/05/2014	21/06/2015	405	405
		GS-5	Seismometer	-38.9944	178.3257	1 348	11/05/2014	25/06/2015	378	378

Table 1

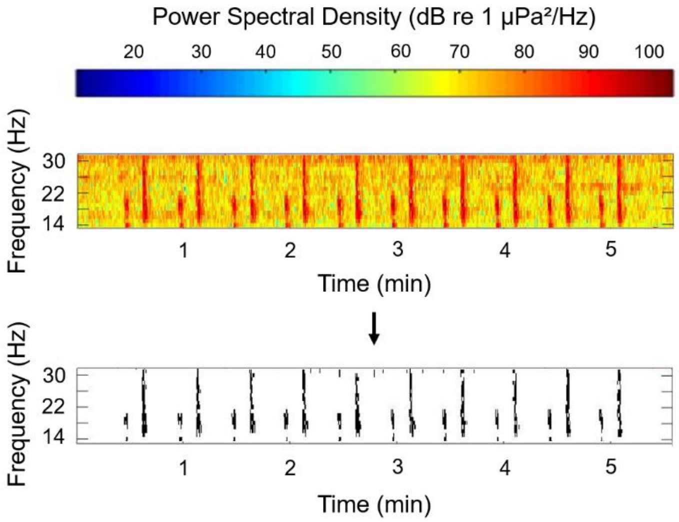
Power Spectral Density (dB re 1 μPa²/Hz)
20 30 40 50 60 70 80 90 100
Frequency (Hz)
30
22
14
1 2 3 4 5
Time (min)
Frequency (Hz)
30
22
14
1 2 3 4 5
Time (min)

Fig 2

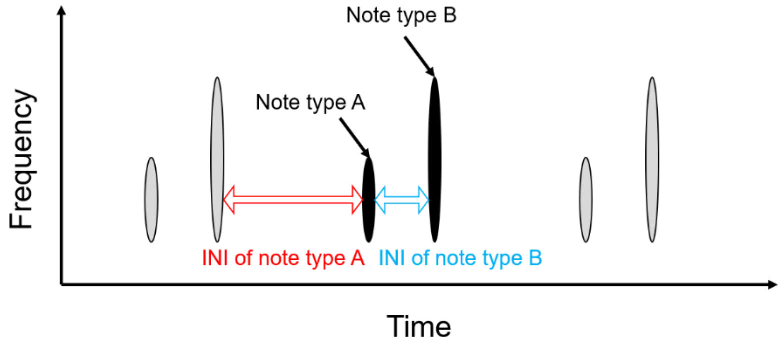
Note type B
Note type A
Frequency
INI of note type A
INI of note type B
Time

Fig 3

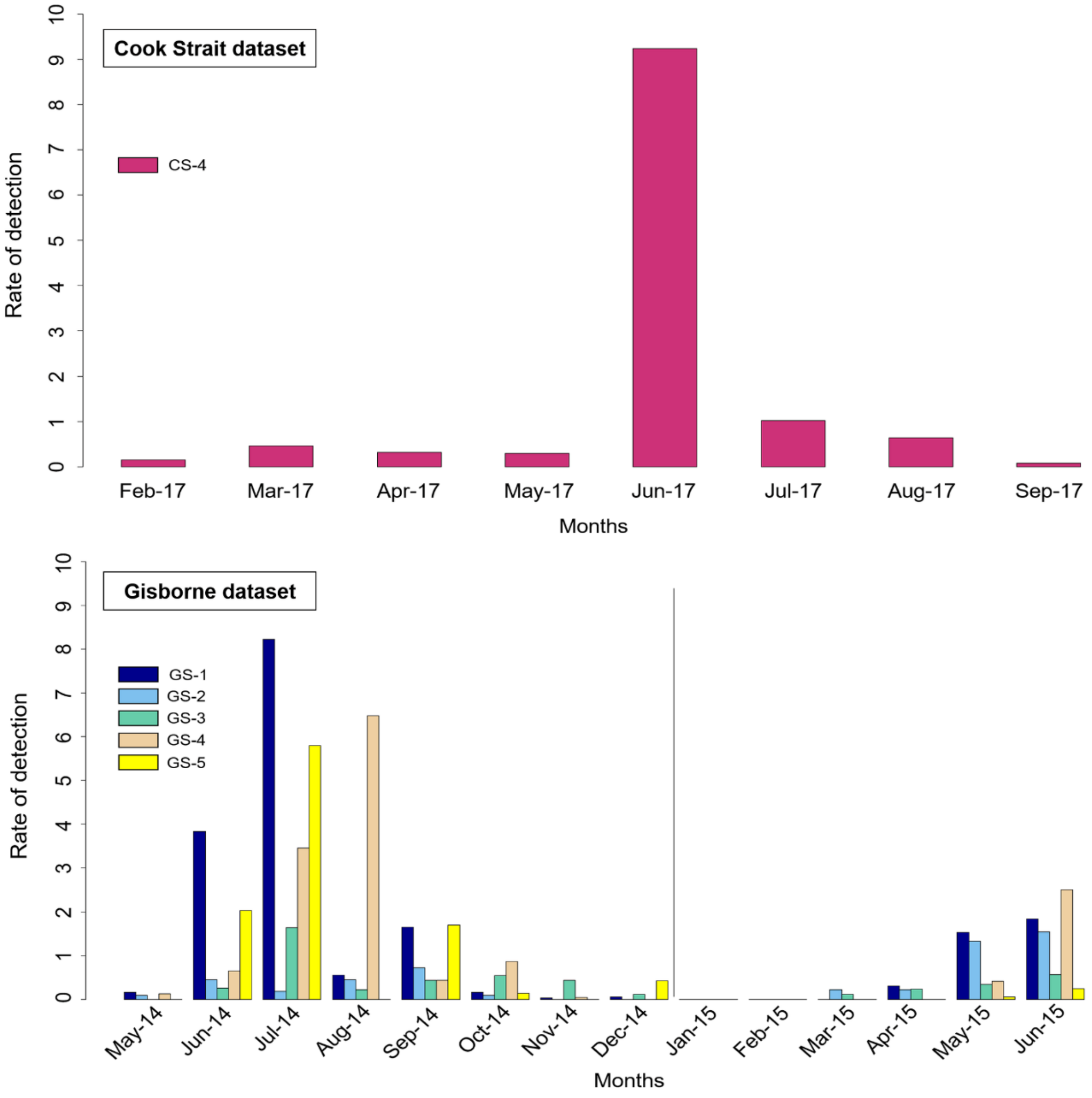

Fig 4

		Total number of fin whales 'doublet' calls detected	Timing of first detection - timing of last detection (DD/MM/YYYY)	Note type A		Note type B	
				Mean peak frequency (Hz)	Standard deviation	Mean peak frequency (Hz)	Standard deviation
Cook Strait	CS-4	529	15/02/2017 - 05/09/2017	20.1	1.04	22.4	0.81
Offshore Gisborne	GS-1	793	27/05/2014 - 16/06/2015	20.6	1.64	21.8	0.79
	GS-2	229	17/05/2014 - 19/06/2015	20.3	0.64	22.2	1.16
	GS-3	211	14/06/2014 - 15/06/2015	19.9	0.94	22.5	0.83
	GS-4	647	25/05/2014 - 18/06/2015	20.6	0.67	22.0	1.25
	GS-5	449	19/06/2014 - 17/06/2015	20.2	0.83	22.3	1.22

Table 2

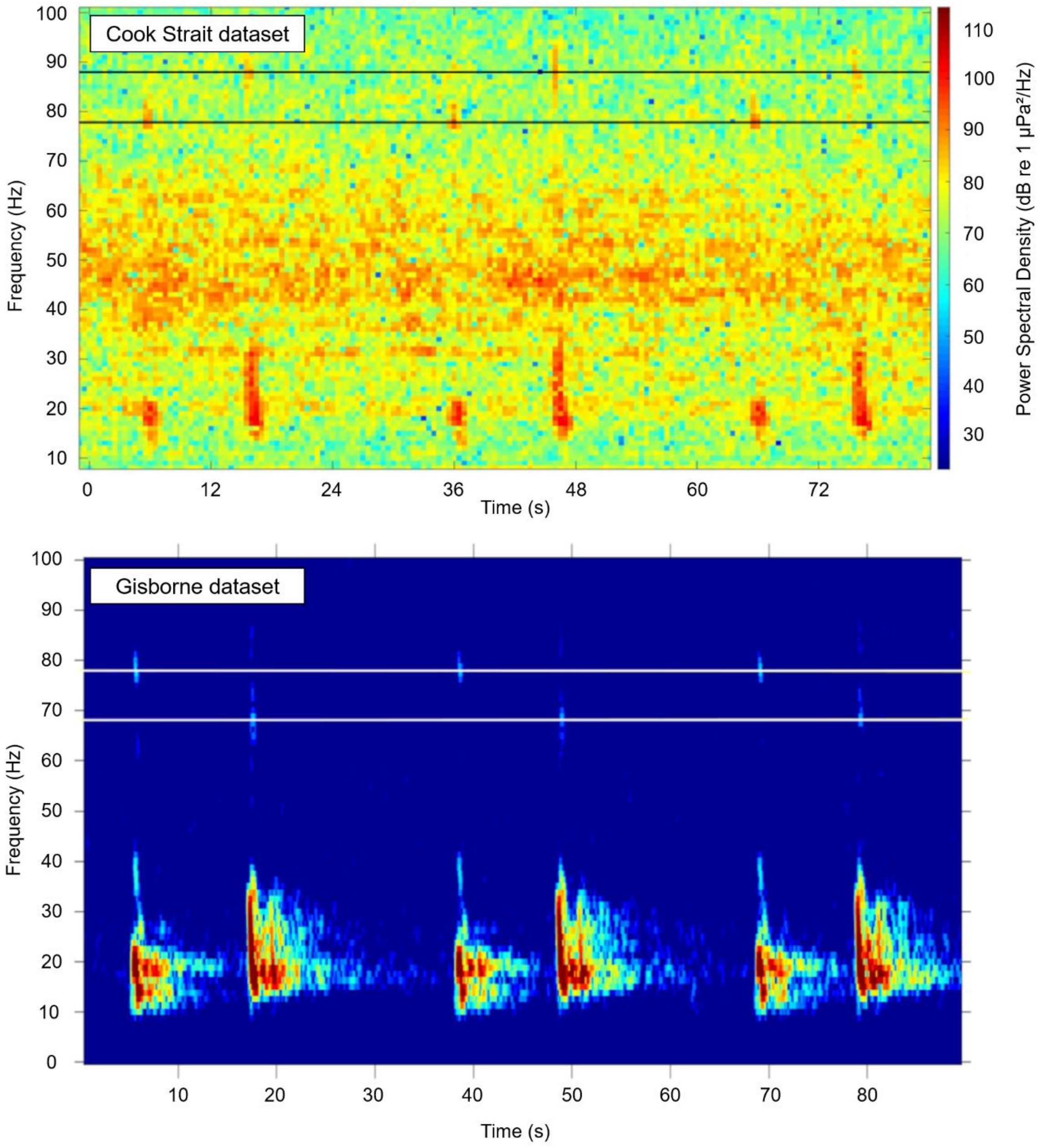

Fig 5

		Number of HF components analyzed	1st component		2nd component	
			Mean peak frequency (Hz)	Standard deviation	Mean peak frequency (Hz)	Standard deviation
Cook Strait	CS-4	232	77.6	1.36	88.2	2.32
Offshore Gisborne	GS-1	204	78.4	1.85	67.8	1.72
	GS-2	41	77.6	2.73	66.4	2.15
	GS-3	12	76.8	2.32	67.0	3.03
	GS-4	174	76.0	2.45	67.4	1.85
	GS-5	196	77.8	2.14	68.8	1.60

Table 3

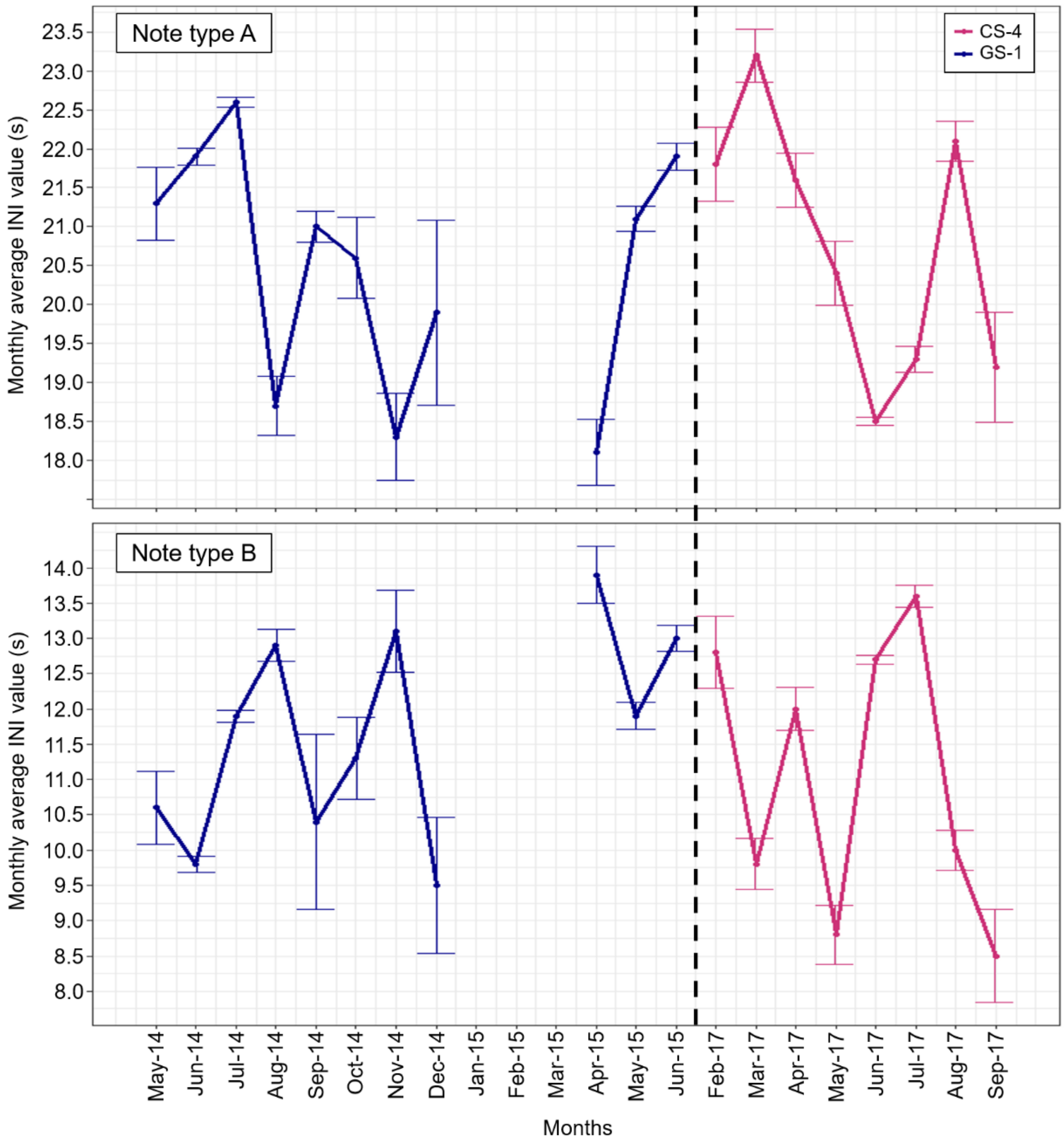

Fig 6

		Total number of fin whales' 'doublet' calls detected	Timing of first detection - timing of last detection (DD/MM/YYYY)	Note type A		Note type B	
				Mean INI (s)	Standard deviation	Mean INI (s)	Standard deviation
Cook Strait	CS-4	529	15/02/2017 - 05/09/2017	20.8	1.66	11.0	1.98
Offshore Gisborne	GS-1	793	27/05/2014 - 16/06/2015	20.5	1.54	11.5	1.74
	GS-2	229	17/05/2014 - 19/06/2015	20.1	1.96	10.8	1.60
	GS-3	211	14/06/2014 - 15/06/2015	21.3	1.98	10.5	2.01
	GS-4	647	25/05/2014 - 18/06/2015	20.9	1.34	10.2	1.49
	GS-5	449	19/06/2014 - 17/06/2015	20.2	1.46	10.3	1.78

Table 4

Location	Longitude	Years	INI (s)	HF (Hz)	Reference
New Zealand	174-176E and 178.5-179E	2016-17 and 2014-15	~20.7, ~10.5	~80, ~90 and ~70, ~80	This study
East Antarctica	65E	2003-4	~14	99	Širović et al., 2009
West Antarctic Peninsula	50-65W	2003-4	~14	89	Širović et al., 2009
Southern Kerguelen Plateau	81E and 75E	2005	~17, ~8	99	Gedamke, 2009
Tasmania	145E	2006-7	~18, ~10	82, 94	Gedamke, 2009
West Australia	115E	2004-7	~27, ~13	99	Gedamke, 2009
Dumont d'Urville	141E	2007	~10, ~8	82, 94	Gedamke, 2009
Juan Fernandez	79W	2016	~14	85.3	Buchan et al., 2019
South Australia	115E, 141E and 153E	2009-17	~28, ~15; ~33, ~17; ~17, ~9	NA	Aulich et al., 2019
Great Barrier Island, New Zealand	176E	1997	17, 9	NA	McDonald, 2006

Table 5

		Reality	
		Presence	Absence
Detection	Detected	True Positive (TP)	False Positive (FP)
	Not detected	False Negative (FN)	True Negative (TN)

S1 Table 1

Trial	Binarization threshold (dB)	Cross-correlation threshold	True Positive (TP)	False Negative (FN)	True Negative (TN)	False Positive (FP)
1	83	0.60	30 %	70%	100 %	0 %
2	83	0.50	75 %	25 %	100 %	0 %
3	73	0.50	0 %	100 %	100 %	0 %
4	88	0.50	89 %	11 %	100 %	0 %
5	78	0.50	0 %	100 %	100 %	0 %
6	93	0.50	55 %	45 %	100 %	0 %
7	63	0.30	0 %	100 %	100 %	0 %
8	88	0.60	67 %	33 %	100 %	0 %
9	93	0.60	29 %	71 %	100 %	0 %
10	73	0.20	100 %	0 %	80 %	20 %

S1 Table 2

Trial	Binarization threshold (relative dB)	Cross-correlation threshold	True Positive (TP)	False Negative (FN)	True Negative (TN)	False Positive (FP)
1	-80	0.60	14 %	86 %	100 %	0 %
2	-100	0.60	15 %	85 %	100 %	0 %
3	-100	0.50	87 %	13 %	94 %	6 %
4	-100	0.55	46 %	54 %	100 %	0 %
5	-90	0.50	100 %	0 %	62 %	38 %
6	-100	0.30	100 %	0 %	0 %	100 %
7	-120	0.40	1 %	99 %	100 %	0 %
8	-160	0.50	0 %	100 %	100 %	0 %
9	-140	0.50	0 %	100 %	100 %	0 %
10	-120	0.50	0 %	100 %	100 %	0 %

S1 Table 3

CS-4					
Presence			Absence		
Detected	TP	86 %	Detected	FP	0 %
Not detected	FN	14 %	Not detected	TN	100 %

S1 Table 4

	CS-4
	N = 250
Precision $P = \frac{TP}{TP + FP}$	100 %
Recall $R = \frac{TP}{TP + FN}$	86 %
Accuracy $A = \frac{TP + TN}{N}$	74 %

	CS-4
β	F-score $F = \frac{(1 + \beta^2)P * R}{\beta^2 P + R}$
2	0.88
1.5	0.90
1	0.92
0.5	0.97

S1 Table 5

	Presence		Absence	
GS-1	Detected	TP = 87 %	Detected	FP = 0 %
	Not detected	FN = 13 %	Not detected	TN = 100 %
	Presence		Absence	
GS-2	Detected	TP = 80 %	Detected	FP = 0 %
	Not detected	FN = 20 %	Not detected	TN = 100 %
	Presence		Absence	
GS-3	Detected	TP = 86 %	Detected	FP = 0 %
	Not detected	FN = 14 %	Not detected	TN = 100 %
	Presence		Absence	
GS-4	Detected	TP = 89 %	Detected	FP = 0 %
	Not detected	FN = 11 %	Not detected	TN = 100 %
	Presence		Absence	
GS-5	Detected	TP = 89 %	Detected	FP = 0 %
	Not detected	FN = 11 %	Not detected	TN = 100 %

S1 Table 6

	GS-1	GS-2	GS-3	GS-4	GS-5
	N = 250				
P	100 %	100 %	100 %	100 %	100 %
R	87 %	80 %	86 %	89 %	89 %
A	75 %	73 %	74 %	76 %	76 %

		GS-1	GS-2	GS-3	GS-4	GS-5
		F-score				
β	2	0.89	0.83	0.88	0.91	0.91
	1.5	0.91	0.85	0.90	0.92	0.92
	1	0.93	0.89	0.92	0.94	0.94
	0.5	0.97	0.95	0.97	0.98	0.98

S1 Table 7
